# Adaptive feature detection from differential processing in parallel retinal pathways

**DOI:** 10.1371/journal.pcbi.1006560

**Published:** 2018-11-20

**Authors:** Yusuf Ozuysal, David B. Kastner, Stephen A. Baccus

**Affiliations:** 1 Department of Electrical Engineering, Stanford University, Stanford, CA, United States of America; 2 Neuroscience Program, Stanford University, Stanford, CA, United States of America; 3 Department of Neurobiology, Stanford University, Stanford, CA, United States of America; Eberhard Karls Universitat Tubingen, GERMANY

## Abstract

To transmit information efficiently in a changing environment, the retina adapts to visual contrast by adjusting its gain, latency and mean response. Additionally, the temporal frequency selectivity, or bandwidth changes to encode the absolute intensity when the stimulus environment is noisy, and intensity differences when noise is low. We show that the On pathway of On-Off retinal amacrine and ganglion cells is required to change temporal bandwidth but not other adaptive properties. This remarkably specific adaptive mechanism arises from differential effects of contrast on the On and Off pathways. We analyzed a biophysical model fit only to a cell’s membrane potential, and verified pharmacologically that it accurately revealed the two pathways. We conclude that changes in bandwidth arise mostly from differences in synaptic threshold in the two pathways, rather than synaptic release dynamics as has previously been proposed to underlie contrast adaptation. Different efficient codes are selected by different thresholds in two independently adapting neural pathways.

## Author summary

In neural circuits, multiple computations are often produced simultaneously by multiple nonlinear mechanisms, making it difficult to assign specific functions to specific neural components. To overcome this barrier in the retina, we used a computational model that reveals internal nonlinear processing. Adaptation to the variance of a signal entails multiple separate properties and mechanisms, one aspect of which is to change the temporal response from more integrating to more differentiating. This adaptive change in temporal bandwidth is known to improve information transmission when the noise level changes. Using the model and pharmacological manipulation, we show that this change in temporal bandwidth can be explained by different thresholds in two parallel neural pathways, rather than properties of vesicle release as have previously been proposed. Our findings provide a rare example where one can disentangle and assign specific computational properties to specific circuit elements in an adaptive circuit.

## Introduction

Sensory systems have the task of encoding stimuli in a changing environment. Neural circuits meet this challenge by changing their neural code in a number of ways that can be explained theoretically by principles of efficient coding. Many sensory environments are composed of strong signals, including high luminance, high contrast, fast velocity or loud auditory stimuli. In each of these cases, in order to use a cell’s dynamic range more efficiently, sensory neurons adapt to the strong stimulus in multiple ways [1–4].

Four distinct changes in the neural code during contrast adaptation have been described in sensory neurons, including in the vertebrate retina, fly visual system and the auditory cortex [2,5–9]. Adaptation to stimulus variance changes the sensitivity, the mean response level or offset, the delay of the response, and finally the temporal frequency preference, or bandwidth. With respect to temporal bandwidth, in natural signals, low spatial and temporal frequencies are predominant, and thus nearby points in space and time have similar intensity. As such, when the signal to noise ratio (SNR) is low, it becomes more efficient to discard weaker and noisier high frequency signals, integrating over the noise across a larger interval of space or time. At high SNR, however, cells can take advantage of the less noisy environment to reduce correlations in the input, and thus the temporal response becomes faster and more differentiating [2,5–9]. This more differentiating response preferentially encodes changes in intensity instead of the absolute intensity. These principles also account for changing spatial receptive fields, explaining why during low luminance, receptive field centers are larger and surrounds are weaker [5,9–12]. A change in temporal bandwidth during contrast adaptation has been observed in the vertebrate retina [1,7,13], and also in human perception [14]. Although this process has been described quantitatively, and its functional importance is understood, how this change in temporal bandwidth is generated within the retinal circuit is not well described.

One obstacle to understanding the sources of these adaptive properties is that they involve nonlinear dynamic properties of the system. A second complication is that signals in the retina are merged through multiple neural pathways, each potentially with its own adaptive properties. A third challenge is that multiple changes in the neural code occur together, making it difficult to understand if any of the changes have specific mechanisms not common to other changes in encoding. Thus is it difficult to gain insights into the interactions of neural components without the use of a computational model. Models have played a key role in the understanding of how nonlinear dynamical systems perform computations, including in single cell processes such as phototransduction [15] and action potential generation [16]. There have been few examples, however, of an understanding of how components of a neural circuit perform specific aspects of nonlinear dynamic computations such as contrast adaptation.

Depletion of synaptic vesicles is thought to be a key source of contrast adaptation, both in terms of a change in gain, offset [17,18] and temporal processing [19,20]. It is unknown however, how parallel pathways interact to influence any of the four adaptive properties.

Here we analyze On-Off amacrine and ganglion cells, which we find to have strong adaptive changes in their preferred temporal feature with contrast. We report a specfic dissociation between the four adaptive properties that requires the On pathway, in that blocking the On pathway abolishes the shift in temporal bandwidth, but leaves changes in gain, offset and the speed of response virtually intact. Our analysis first concludes that the shift in bandwidth with contrast arises from a changing ratio of activation of the On and Off pathway. Thus the temporal derivative that is computed during high contrast is largely a difference between two neural pathways.

To understand the source of this differential activation of neural pathways in the intact system without pharmacological manipulation, we analyzed a previously reported computational model that captures all adaptive properties of On-Off ganglion cells to changing contrast [20]. This model consists of a linear temporal filter, a time-independent or static nonlinearity that applies a threshold and saturation to the input, and a first-order kinetic system that creates the dynamic adaptive changes in response to a changing input [21,22]. This linear-nonlinear-kinetic model (LNK) captures all adaptive properties in an environment of changing contrast as well as the membrane potential response nearly to within the variability of the cell. Although other models have captured the properties of fast adaptation in ganglion cell membrane potential or firing rate [23–25], these models do not include the slow adaptation seen in ganglion cells, and thus would not capture all adaptive properties observed here. Given the known properties of the retina, in the LNK model, the nonlinearity corresponds to the threshold at the bipolar cell synaptic terminal, and the kinetic system captures the dynamics of synaptic vesicle release [20].

To assess why the two pathways adapted differently to contrast, we analyzed differences in the computational components of the two pathways. One might expect that because adaptive properties have been assigned to the kinetics block that differential adaptation must come from different adaptive kinetics in the On and Off pathways, representing differential properties of vesicle release. However, we find that the different thresholds in the two pathways are the primary cause of the change from the more integrating to more differentiating response, rather than the dynamics of synaptic release as has previously been proposed [18,20]. These results reveal a picture where different components of adaptation are produced by different mechanisms. At the level of the ganglion cell membrane potential, changes in gain, offset and the speed of temporal processing are produced by synaptic depression [20]. But to create the additional change from a more integrating to differentiating response, the difference in threshold in the two pathways leads to different levels of adaptation in the two pathways. The overall result is that increased contrast changes the mixture of the two pathways. By analyzing a model whose computational components can be mapped to neural components, different rules of efficient coding can be assigned to different internal computations and mechanisms in a parallel neural system.

## Results

**Fig 1:**
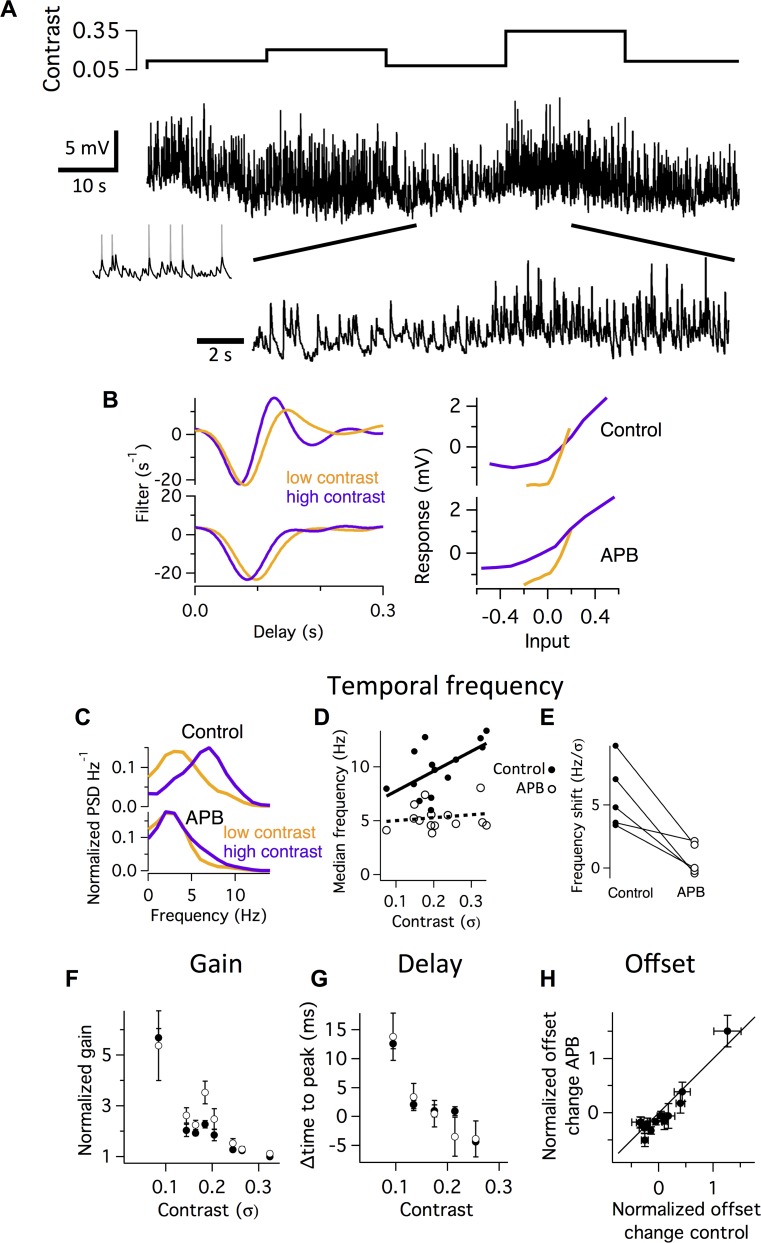
The On pathway is specifically required for temporal bandwidth adaptation. **A.** Top. Contrast of a randomly flickering stimulus drawn from a Gaussian intensity distribution with a constant mean. Contrast values ranging from 3–35% were each presented to the retina for periods of 20 s. Middle. Example membrane potential recording lasting 100 s of a ganglion cell responding to the 5 different contrast levels shown. Spikes were digitally removed (inset shows segment of raw recording). Bottom. Expanded segment showing transition between higher and lower contrast. **B.** Linear filters (Left) and nonlinearities (Right) fit during low and high contrast to membrane potential recordings in control solution (top) and with 30 μM APB (bottom). Filter amplitudes are normalized, with gain represented in the slope of the nonlinearity, and have units of s^-1^ so that the convolution over time of the filter with a dimensionless stimulus yields a dimensionless output. **C.** Temporal power spectrum of the response at high and low contrasts in the control (top) and APB (bottom) conditions for an example cell. **D.** Median frequency computed as the half-maximal value of the cumulative distribution function of the power spectrum as a function of contrast in the control and APB conditions for one cell. Lines indicate a linear fit. **E.** Shift in the median temporal frequency as a function of contrast, computed as the slope of the line in (**D**) for five cells, shown in the control and APB conditions. **F.** Gain of cells as a function of contrast, measured as the average slope of the nonlinearity, averaged over 5 cells. **G.** Delay in response, measured as the time to the first negative peak of the filter as a function of contrast. **H.** Offset in response measured as the mean membrane potential in 4 s segments, normalized by the standard deviation and computed during all contrasts and compared between the control and APB conditions.

A randomly flickering visual stimulus was presented from a standard cathode ray tube to the isolated salamander retina. The stimulus was spatially uniform and white in color with an intensity that changed every 30 ms, and was drawn from a Gaussian distribution with a constant mean. Every 20 s, the temporal contrast changed to one of 15 contrasts by varying the standard deviation of the distribution. We recorded the intracellular membrane potential response from On-Off amacrine and ganglion cells for at least 300 s (Fig 1A, S1 Fig). Spikes were digitally removed for analysis of the subthreshold membrane potential [20].

To measure the influence of the On neural pathway to contrast adaptation, we presented the stimulus in a control condition, and while blocking the On pathway using L-AP4 (APB), a metabotropic glutamate receptor agonist that blocks synaptic input to On bipolar cells.

To quantify different adaptive properties at different contrasts, we used the standard approach of computing a linear nonlinear (LN) model, consisting of a temporal filter that represents the average feature conveyed by a neuron, and a static, or time-independent nonlinear function that captures threshold, gain and any saturation [7]. An LN model that changes with different stimulus statistics has served as a definition of an adaptive system. In this case, the purpose of fitting an LN model is as a statistical summary of the encoding properties of the system to reveal that the system is adaptive and therefore more complex than an LN model, and it is not assumed that the LN model will capture the system under all conditions. In fact, an LN model that was accurate across different stimulus statistics would imply the system was not adapting. We found that the average gain of a cell, represented by the slope of the nonlinearity, decreased with contrast both in the control and APB conditions, with little difference in the two conditions (Fig 1B and 1C). We quantified the speed of the response as the delay until the trough of the linear filter, which changed with contrast as expected. APB also did not influence the contrast-dependent change in the delay of the response (Fig 1D). We further compared the offset of the response by computing the mean membrane potential at different times during the recording, and compared them in the control and APB conditions. The On pathway was similarly not required for adaptive changes in the mean membrane potential (Fig 1E).

We then examined the change in temporal bandwidth produced by contrast adaptation by analyzing the linear filter. As previously reported [7,26], the filter at low contrast was more monophasic, and at high contrast the filter was more biphasic (Fig 1B). As a simple alternative means of quantifying this change in the linear filter, we computed the Fourier transform of the filter and examined the filter in the frequency domain at each contrast. The median temporal frequency of the filter was lower at low than at high contrast. In addition, the power at the lowest frequency was greatly attenuated at high contrast, reflecting the more biphasic filter (Fig 1F–1H). We computed the average shift in the median frequency as a function of contrast, which was 5.7 ± 1.2 Hz/σ (n = 5), where σ is contrast units. In APB, the filter changed its temporal frequency much less, shifting 0.7 ± 0.5 Hz/σ, which was significantly less than the control condition (P < 0.02). In total, the effect of the On pathway on adaptation was highly specific, in that only the adaptive shift in temporal bandwidth required both On and Off pathways, whereas changes in gain, response delay and offset did not.

### On-Off cells have greater changes in temporal bandwidth

**Fig 2:**
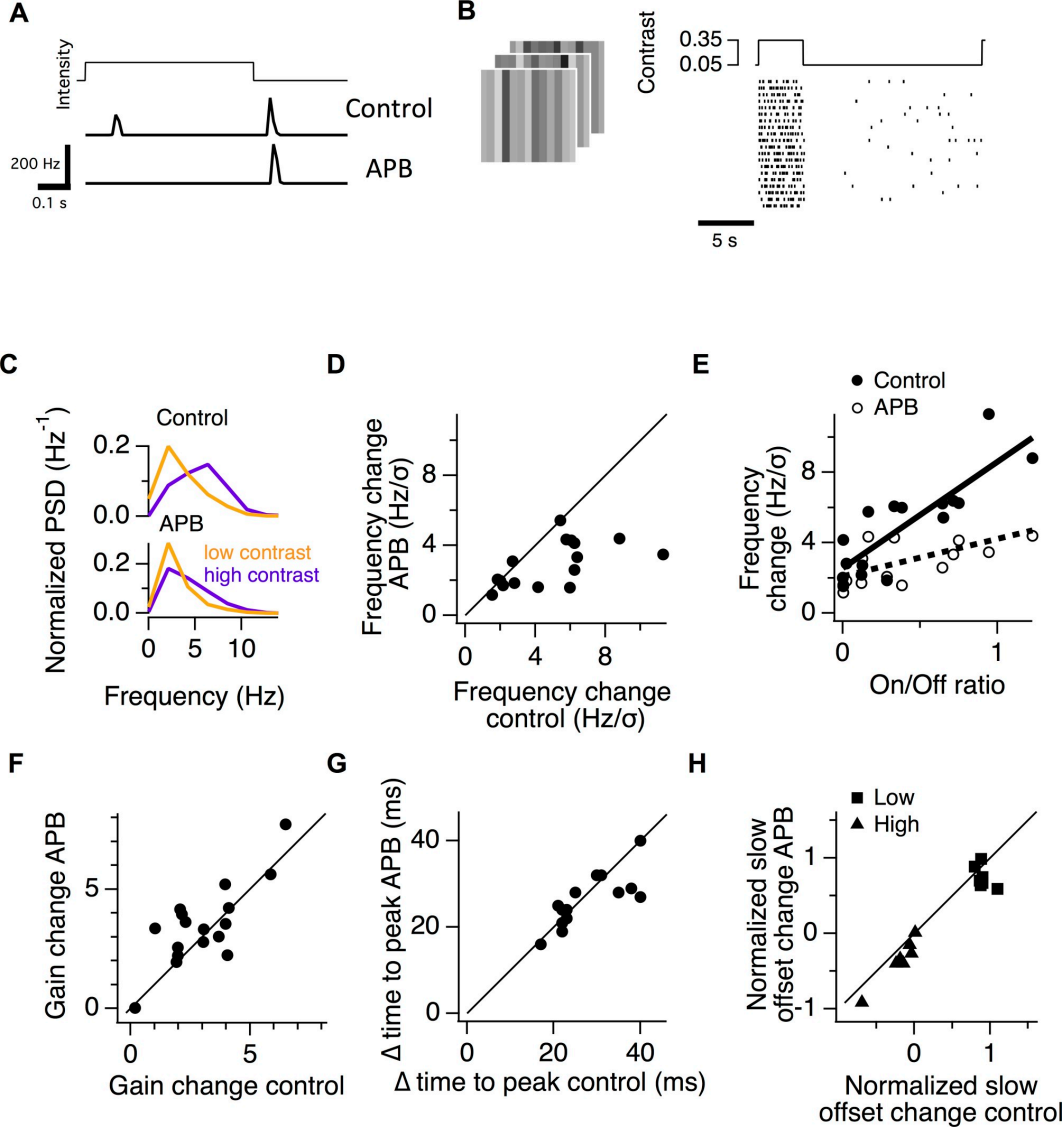
Cells with more On pathway input have a greater shift in temporal frequency. **A.** Peristimulus time histogram of the spiking response of an example On-Off ganglion cell recorded with a multielectrode array, with and without the application of APB. **B.** Example spiking response of a ganglion cell during a one-dimensional spatiotemporal white noise stimulus that alternated between high (35%) contrast for 4 s, and low (5%) contrast for 16 s. In the raster plot, each line is the response for an example cell to a different white noise sequence. **C.** Power spectrum of temporal filters of an example cell for high and low contrast in a control condition (top) and with APB (bottom). The LN model for the cell is shown in (S2 Fig). **D.** The shift in the median temporal frequency of the filter during a change in contrast compared for the control condition and in APB. The shift in temporal frequency is shown divided by the contrast shift of 30%, (from 35% to 5%) to compare with the results of Fig 1. **E.** The change in frequency shown as a function of the On/Off ratio of each ganglion cell for a population of 16 cells. Also shown is this frequency change for the same cells in APB. Lines are linear fits to the data. **F.** Gain change computed as the change in the average slope of the nonlinearity between high and low contrast compared with and without APB. **G.** The change in the time of the first negative peak of the linear filter between high and low contrast, with and without APB. **H.** The slow change in average firing rate taken from a linear fit to the firing rate as a function of time during either the low contrast interval (■) or during high contrast (▲). Changes in rate were normalized by the measured maximum rate in that interval, which could be smaller than the maximum value of the linear fit. Data was only analyzed from cells that adapted during low contrast (n = 7), increasing their firing rate. Cells that sensitized, decreasing their firing rate during low contrast [56] were excluded (n = 5 cells).

We then analyzed the spiking responses of ganglion cells with and without APB that included both On/Off cells and Off cells with no On pathway input, which were recorded with a multielectrode array in response to a spatiotemporal stimulus. We first identified the relative strength of the On and Off pathways using a uniform field flash, and computed the ratio of the firing rate of the On and Off responses (Fig 2A). Then we presented a one-dimensional spatiotemporal stimulus consisting of randomly flickering lines drawn from a Gaussian distribution that alternated between high contrast (4 s) and low contrast (16 s) (Fig 2B). LN models during high and low contrast consisted of a spatiotemporal filter calculated as the spike-triggered average stimulus, followed by a static nonlinearity computed after convolving the entire spatiotemporal filter with the stimulus [27]. To analyze the time course of the response, we extracted the average time course of the spatiotemporal filter as the first principal component of the temporal dimension of the spatiotemporal map (S2 Fig).

As in the case of membrane potential recordings, high contrast shifted the temporal bandwidth to higher temporal frequencies (Fig 2C–2E). Across all cells, APB reduced the shift in frequency from 5.0 ± 0.7 to 2.9 ± 0.3 Hz/σ (n = 16, P < 0.01, paired t-test) for the 0.3 σ change in contrast (Fig 2D and 2E). In addition, we found that cells with the largest changes in temporal bandwidth had the greatest input from the On pathway. We fit a line to the change in temporal bandwidth as a function of On/Off ratio for the population of cells. Across the range of On/Off ratios measured in the population, this linear fit indicated that the cells with the highest On/Off ratio changed their temporal bandwidth by a factor of 3.6 ± 1.3 (s.e.m. by standard bootstrap) greater than Off cells, which had a zero On/Off ratio (Fig 2E). As with membrane potential recordings, APB had little effect on adaptive changes in gain, delay or offset with contrast (Fig 2F–2H). These properties did not change substantially with On/Off ratio (S3 Fig), with the exception of slow changes in offset during high contrast, which decreased for cells with greater On/Off ratio. However, APB had little effect on this relationship.

We further analyzed bipolar cell recordings with the expectation that since they receive input from a single pathway, they would not have large changes in temporal frequency with contrast. Consistent with this idea, although bipolar cells showed changes in gain that are smaller than that of ganglion cells as previously reported [7,28], they did not change their temporal frequency with contrast (S4 Fig).

From both intracellular membrane potential recordings and extracellular recordings, we conclude that cells with strong On and Off input have the highest shift in temporal frequency during contrast adaptation. Furthermore, blocking the On pathway specifically reduces this shift in temporal frequency but not the adaptive properties of changes in gain, delay or offset.

### LNK model captures the separate contribution of On and Off pathways

To consider how one can interpret our pharmacological results, in general, the response of a cell is *F*(*B*_*ON*_)+*G*(*B*_*OFF*_)+*H*(*B*_*ON*_,*B*_*OFF*_), where *F*(*B*_*ON*_) and *G*(*B*_*OFF*_) are the additive contribution from depolarizing and hyperpolarizing bipolar cells, respectively, and *H*(*B*_*ON*_,*B*_*OFF*_) is the contribution from nonlinear interactions between the two pathways. The application of APB reveals input from *G*(*B*_*OFF*_) alone, subtracting both *F*() and *H*(). If there were no interactions between the two pathways (*H*() = 0), then APB could reveal the separate contributions of both Off and On pathways, *F*() and *G*(). In the intact circuit, however there are many potential nonlinear interactions that could occur between On and Off pathways, as numerous types of amacrine cells deliver inhibition from one pathway to another, known as ‘crossover inhibition’ [29]. Thus one cannot without further knowledge simply assume that the two pathways are independent, and subtract the Off pathway under APB from the total response to discover the separate contribution of the ON pathway. However, this possible approach is indicated by the success of a linear-nonlinear-kinetic (LNK) model [20] (described below) with two independent pathways that we used previously to accurately capture the responses of On-Off ganglion and amacrine cells. Like most models of parallel pathways, however, it is difficult to know whether the separate model pathways correspond to separate neural pathways. We thus tested pharmacologically whether the model pathways did in fact capture the separate contributions of the On and Off pathways.

**Fig 3:**
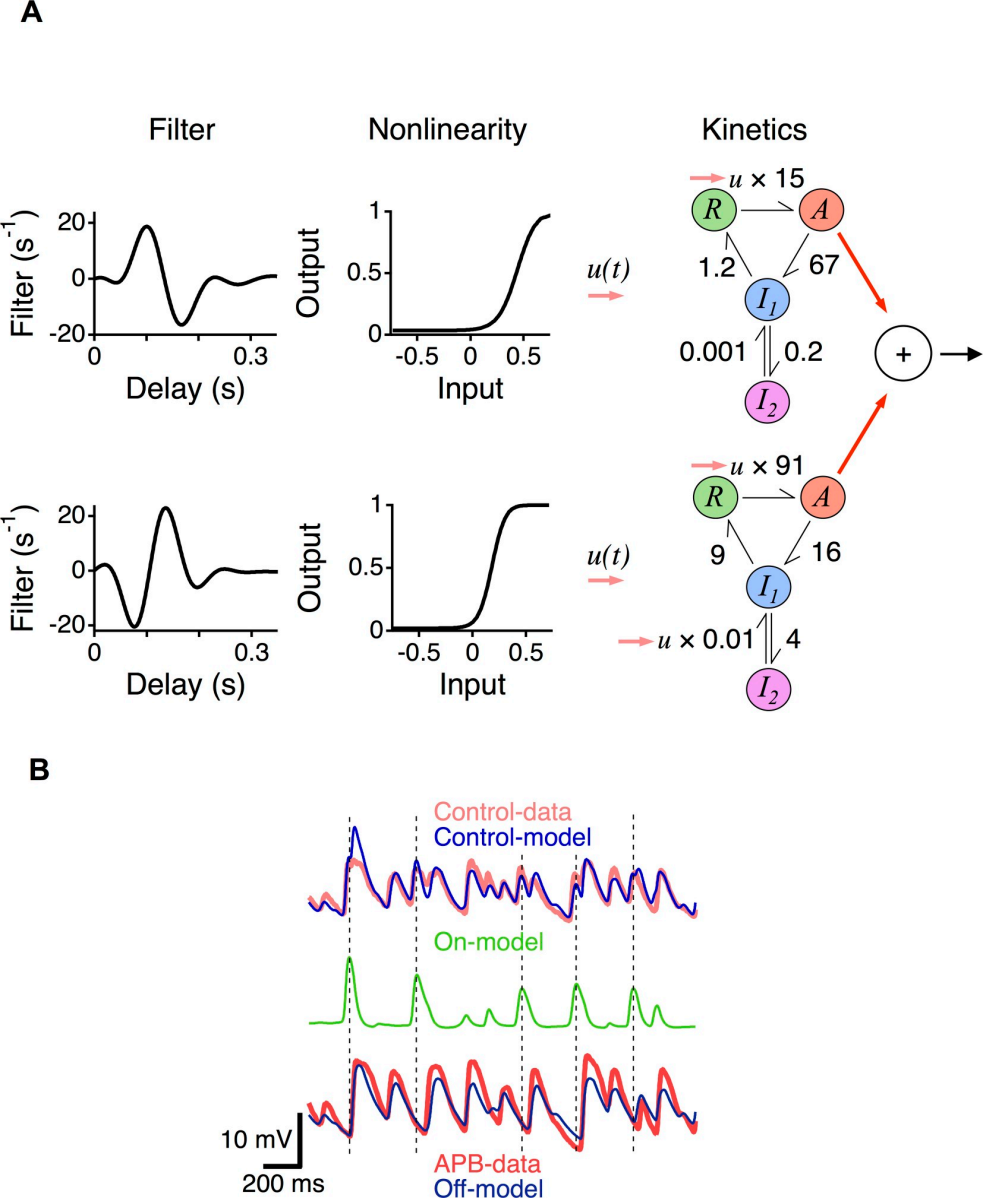
A two-pathway LNK model reveals additive contributions of the On and Off pathways. **A.** Linear–nonlinear–kinetic (LNK) model of an On-Off ganglion cell. Each of two pathways (Top, On pathway; Bottom, Off pathway), consists of a linear filter, a nonlinearity and a kinetics block. **B.** Top. Subthreshold membrane potential recordings from an On-Off amacrine cell under control conditions compared to the total output of the two-pathway LNK model. Middle. The On pathway output in the LNK model. Dotted lines indicate peaks of large responses of the On-pathway model. Bottom. Membrane potential recordings with APB compared with the Off pathway output in the control LNK model.

In each of the two pathways of the LNK model, the first stage consists of a linear temporal filter *F*_*LNK*_, which represents the average response at this intermediate stage to a brief flash of light. The Off pathway contained a filter with a negative first peak, and the On pathway contained a filter that had a more delayed positive first peak (Fig 3A, top). Note that this filter, *F*_*LNK*_ is different from the linear filter, *F*_*LN*_, of an LN model, as *F*_*LN*_ contains all temporal processing for the entire system as opposed to the three part system of the LNK model where dynamics are contributed both by the initial filter *F*_*LNK*_ and the final kinetics stage. Thus the linear filter *F*_*LN*_ is only an approximation to more complex nonlinear dynamics. In the next stage, a static nonlinearity *N*_*LNK*_ applies a threshold and saturation to the response, as well as setting a baseline sensitivity. The final stage consists of a 4-state first order kinetics model, a system that transitions between different states governed by a set of rate constants [30,31]. The output of the nonlinearity scales one or two of the rate constants in the kinetics block. For models fit to amacrine and ganglion cells, these rate constants are similar to previously measured rates of bipolar cell synaptic release [32–34]. In each pathway, the output of the kinetics block is the active state. The final output is the sum of the two independent pathways, meaning that the effects of the two pathways combine additively.

To verify the correspondence of the LNK model to the separate properties of the On and Off pathways, we first fit a two-pathway LNK model to membrane potential responses recorded in a control condition (Fig 3A). The correlation coefficient between the model output and the data was (89 ± 5% n = 4). We then blocked the On pathway pharmacologically using APB, and compared the Off pathway of the control model to a single pathway LNK model fit in the presence of APB (Fig 3B). The correlation coefficient between the Off pathway output in the control case and the model with APB was 96 ± 1% (n = 4). Furthermore, the On pathway of the control model contained fluctuations that were present in the data in the control case, but not when APB was present. This indicates that the drug APB, which selectively suppresses the On pathway, removed the model On pathway without changing the model Off pathway. Thus, despite the combination of two independently adapting pathways, the two-pathway LNK model accurately reveals the separate contributions from those pathways, allowing an analysis of how their components give rise to the overall behavior of the cell. Furthermore, because the LNK model sums its two pathways, and the Off pathway fits the pharmacologically defined Off pathway, we can conclude that under these stimulus conditions the contributions of the On and Off pathways corresponded to the added parallel adaptive pathways represented by the model. This observation also rules out the possibility that saturating the On pathway with APB adds a large steady offset to the Off pathway through crossover inhibition. If this were the case, the model would have been in error because the threshold of the Off pathway would effectively shift under application of APB. Note that this does not rule out inhibitory contributions that cross pathways, but does show that the system can be analyzed as though it adds two separate adapting pathways.

**Fig 4:**
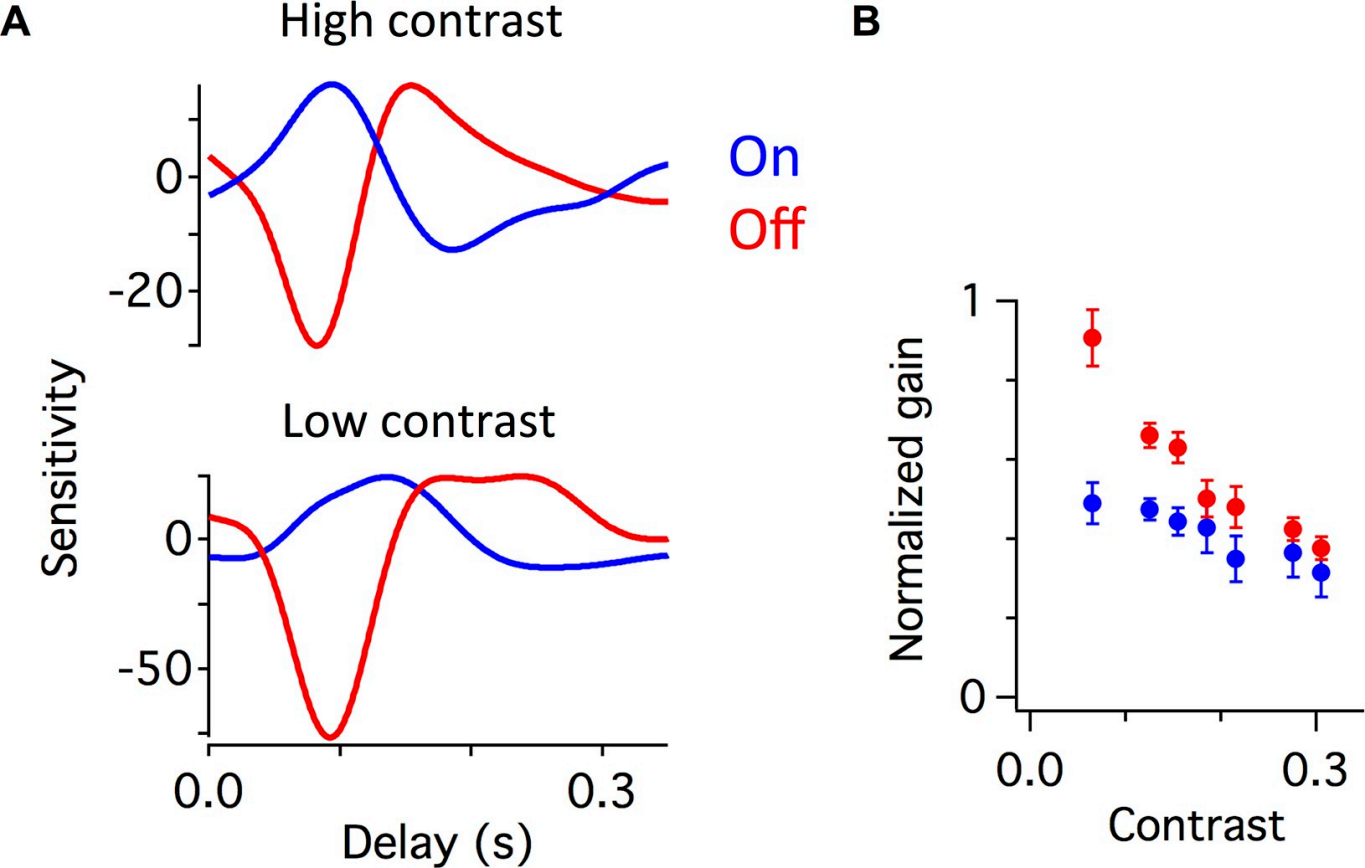
Contrast changes the weighting of On and Off pathways. **A.** Linear filters computed from the On and Off pathway contributions at high contrast (top) and low contrast (bottom). In this representation, the gain is shown as the amplitude of the filter, so that the filter is equal to the normalized filter as shown in Fig 1B times the average slope of the nonlinearity. The Off pathway contribution was measured as the response in the presence of APB, and the On pathway was estimated by subtracting the APB response from the control response. **B.** Normalized gain of the On and Off pathways computed as a function of contrast, measured as the amplitude of the filter in **A.** Each cell’s gain was normalized so that the total gain of the full response averaged over all contrasts was one.

Given that under these conditions the circuit adds the two similar pharmacologically defined pathways, we first analyzed the system by computing the gain of the two pathways from the recorded responses as a function of contrast. To do so, we computed LN models from the response under APB, representing the Off pathway, and from the difference between the control and APB responses, which is the contribution that requires the On pathway. Once again, we computed the gain as the average slope of the nonlinearity. We found that at high contrast, the gain of the On and Off pathways were much more similar, whereas at low contrast, the gain of the Off pathway was much larger than that of the On pathway (Fig 4). This analysis revealed that the two pathways adapted differently to contrast, with the Off pathway adapting more than the On pathway (Fig 4B). Thus, as the contrast increased, different responses in the two pathways yielded a different mixture of the On and Off pathways, creating a more biphasic response than would result from a single pathway. This finding provides an explanation as to how the two pathways generate a more biphasic temporal filter at high contrast.

### Two pathways increase the change in temporal bandwidth

**Fig 5:**
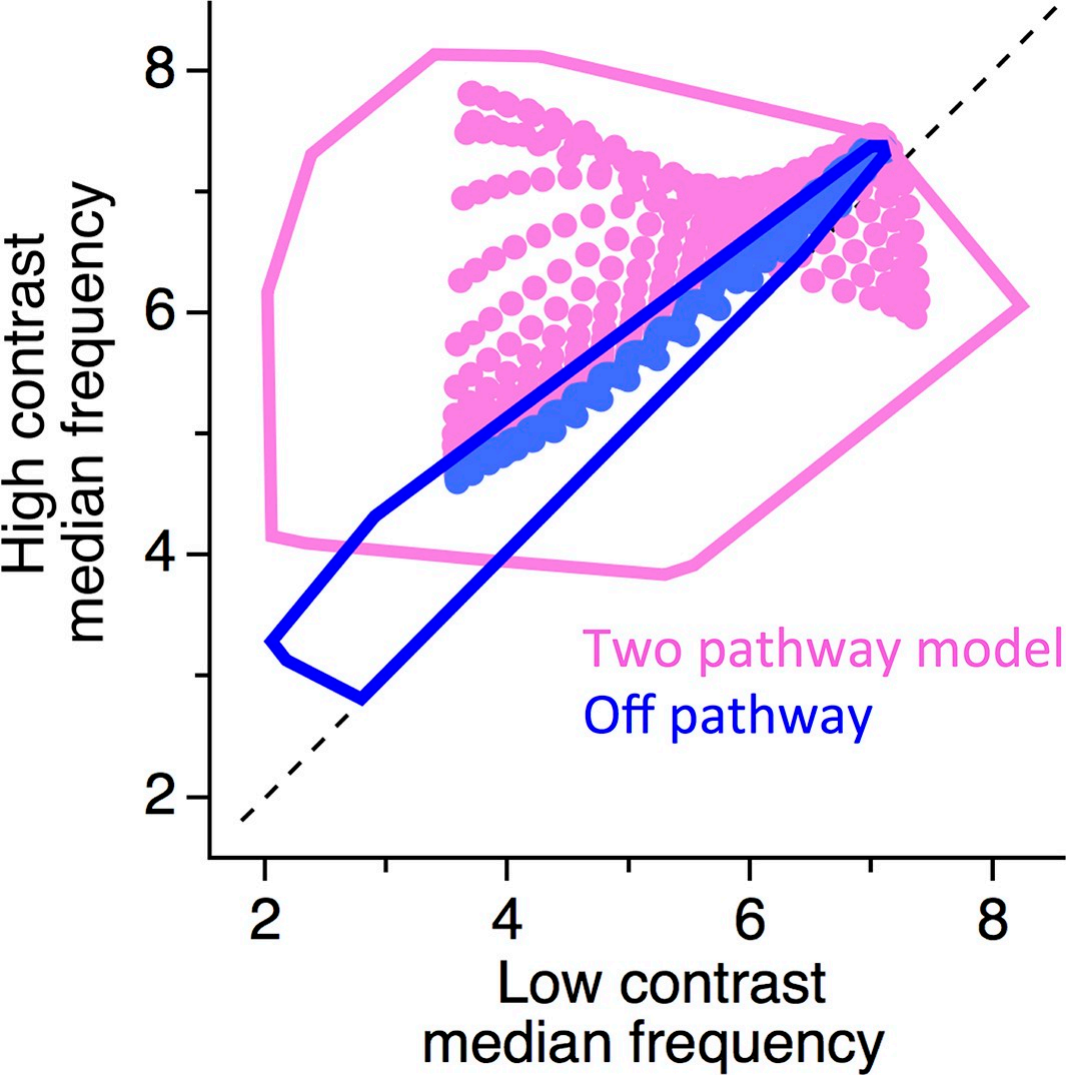
Two LNK pathways can produce greater changes in temporal bandwidth than one pathway. For LNK models for a set of eight cells, two parameters of the Off pathway were varied, the rate of fast inactivation *k*_*fi*_, and the rate of fast recovery, *k*_*fr*_ in a grid search with values ranging between a factor of five greater than or less than the values fit to data. The model contained either the Off pathway alone, or both On and Off pathways. On pathway parameters were not varied. Shown are the median temporal frequencies of the model at high and low contrast for a linear filter fit to the output of the entire model. Symbols indicate values for one cell when parameters were varied. Lines indicate the convex hull of all points across all cells.

To explore the range of bandwidth adaptation allowable by one or two neural pathways, we varied the model parameters of the kinetics block of the Off pathway alone (Fig 5). We assessed the contrast-dependent change in temporal frequency achievable with the Off pathway alone, or when an On pathway was added whose parameters were not varied. We focused on two rate constants, the rate of fast inactivation, *k*_*fi*_, and the rate of fast recovery, *k*_*fr*_, based on previous studies that these parameters strongly influence the change in temporal bandwidth and temporal filtering [20]. For each cell, we varied these two parameters in the Off pathway in a grid search over a factor of 25 around their control values, which were taken from fits to the data recorded intracellularly. We found that when the Off pathway was tested alone, varying the parameters had a strong effect on the mean (baseline) frequency response. However, varying these parameters only caused a modest change in the level of adaptation, ranging from no shift in frequency to a maximum of a ~25% shift in frequency when the contrast was changed (Fig 5). However when the two-pathway model was used, the span of behaviors was much greater, as some parameter sets caused the median frequency to increase by more than a factor of two at high contrast. In addition, it was possible for some parameter sets to substantially decrease the median frequency at high contrast. A prediction of the model is therefore that Off cells will have a small amount of temporal frequency adaptation, and cells with a greater contribution of the On pathway will have greater temporal frequency adaptation. Consistent with this prediction, cells with a zero On /Off ratio (Off cells), still have a small frequency shift that is not affected by APB, and cells with a greater On/Off ratio have greater temporal frequency adaptation (Fig 2E). Thus, the combination of two pathways could create a greater shift in temporal frequency than could be achieved with a single pathway.

### Adaptive change in stimulus feature through differential processing in two pathways

**Fig 6:**
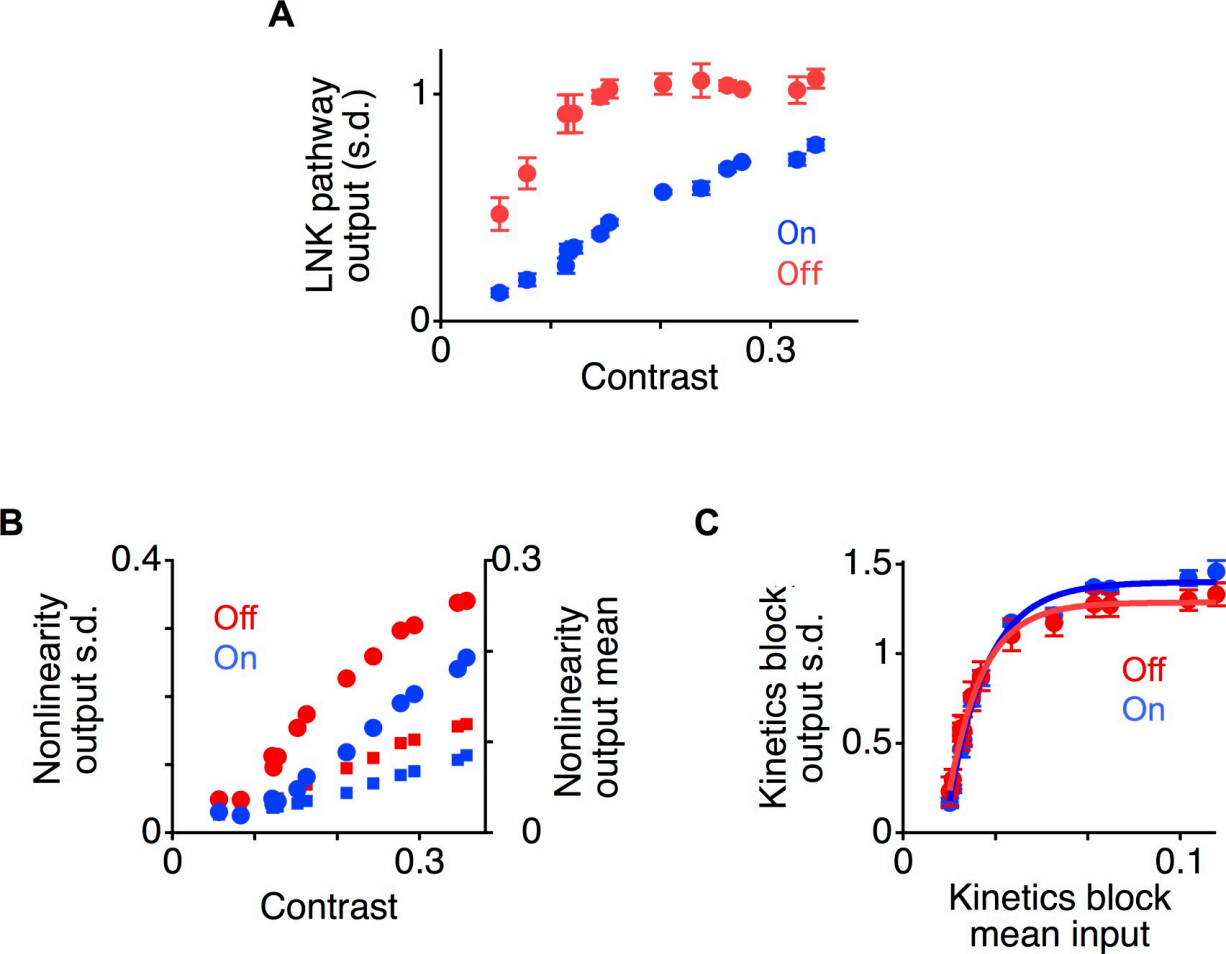
Differential adaptation in On and Off pathways. **A.** Standard deviation for the On and Off pathway outputs of LNK models of eight amacrine and ganglion cells as a function of contrast after normalizing the standard deviation of the entire output to one. Numbers do not sum to one because signals from the On and Off pathways partially cancel. **B.** Standard deviation (circles) and mean (squares) of the nonlinearity output *u*(*t*) in each pathway as a function of contrast. **C.** Standard deviation of the kinetics block output *A*(*t*) in each pathway as a function of the mean input to the kinetics block 〈*u*〉. A canonical nonlinearity *N*_0_ with a threshold at zero was used to generate *u*(*t*) as a function of contrast.

What internal computational and mechanistic properties of the two pathways underlie this differential adaptation to contrast? To answer this question, we analyzed the components of the two-pathway LNK model. We first quantified the relative contribution of the two pathways by computing the standard deviations of the output On and Off pathways at different contrasts. At low contrast, the On pathway contributed less to the total standard deviation of the recording (On, 22 ± 3%, Off 78 ± 12%, n = 8), whereas at high contrast, the contributions of the two pathways were more similar (42 ± 2% for On and 58 ± 2% for Off). Between the extremes of low and high contrast, we then examined in greater detail how the magnitude of each pathway changed as a function of contrast. As the contrast increased, first the output amplitude of the Off pathway increased more rapidly than that of the On pathway (Fig 6A). Then the amplitude of the Off pathway reached a plateau, which indicated that adaptive gain changes in the Off pathway were compensating for the increased input. Consistent with the analysis of the data using LN models (Fig 4), the output of the On pathway steadily increased throughout the contrasts tested, indicating that less adaptation occurred with increased contrast.

### Different thresholds have a greater effect on differential processing than different adaptive kinetics

By definition, an LN model is a non-adapting system, whereas an LNK system shows adaptation. Therefore, one might think that differential adaptation would naturally be caused by differences in the parameters of the kinetics block in the two pathways. However, the adaptive properties of an LNK pathway are a result of both the nonlinearity and the kinetics block [20]. We thus analyzed how each stage of the model in the two pathways contributed to the differential change in output magnitude, and thus the adaptive change in temporal differentiation. Because the linear filters in the two pathways were normalized in amplitude, and the mean of the input is constant, the output amplitude of the first stage in each pathway simply reflects the contrast. Thus no differential processing occurs at this stage except for the difference in the preferred stimulus feature.

We measured how the output magnitude of the nonlinearity varied as a function of its input in the two pathways. Comparing the two nonlinearities, the On pathway had a higher threshold as previously reported for salamander On-Off ganglion cells [35], and a more shallow slope than the Off pathway as seen in mammalian retina [36,37] (Fig 5). Due to its lower threshold, the output of the Off nonlinearity increased at a lower contrast than in the On pathway (Fig 6B). However, as contrast increased, because of the steeper slope in the Off nonlinearity, the Off output then began to rise at a slower rate than the On pathway. In comparison, the output of the On pathway rose steadily with contrast above threshold.

To compare the independent contribution of the kinetics block in each pathway, we presented a standardized input *u*(*t*) to the two kinetics blocks, and measured in each pathway the magnitude of the output *A*(*t*), the active state. Previously it was shown that because of the threshold nonlinearity, an increase in contrast causes an increase in both the mean and standard deviation of *u*(*t*). However, only the mean 〈*u*〉 controls adaptation in the kinetics block, thereby controlling the standard deviation of the output *A*(*t*) [20]. Thus we computed the amplitude of the kinetics block output as a function of the mean input 〈*u*〉. To generate *u*(*t*), we used a nonlinearity *N*_0_ with a threshold at zero for both kinetics blocks. This caused both the mean and standard deviation of *u*(*t*) to increase linearly with contrast. When 〈*u*〉 increased, the standard deviation of the kinetics block output *A*(*t*) increased quickly at first, but then increased with a decreasing rate as the kinetics block adapted (Fig 5C). Comparing the two pathways, the standard deviation of the kinetics block as a function of the mean input 〈*u*〉 was similar. The Off pathway, however, adapted slightly more than the On pathway in that on average the standard deviation of the On pathway increased only 1.22 ± 0.04 (n = 8) times more than the Off pathway across different mean inputs. Based on this separate analysis of the different stages of processing, differences in the output of the two pathways with contrast appeared to be caused more by the different nonlinearities than by the different kinetics blocks.

**Fig 7:**
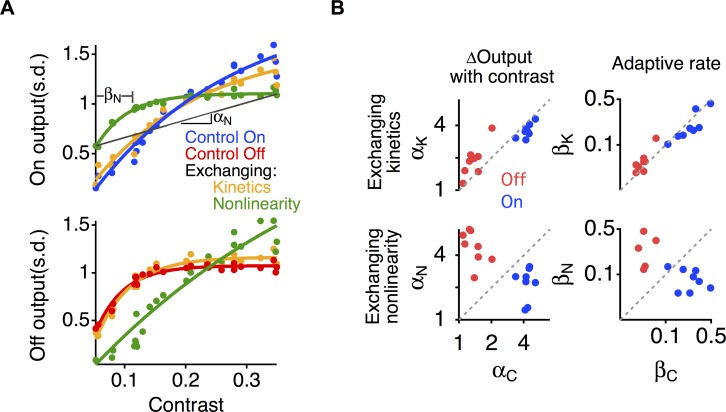
Differences in threshold create differential adaptation. **A.** Output vs contrast for the On (top) and Off (bottom) pathways of an On-Off amacrine cell, compared with the same curves after exchanging the nonlinearities or the kinetics blocks of the two pathways. Diagonal line labeled α_*N*_ indicates the average slope of the output vs contrast curve when the nonlinearities were exchanged. Point labeled β_*N*_ indicates the adaptation rate constant as a function of contrast when the nonlinearities were exchanged, computed from an exponential fit to the curve. **B.** The values for α (left) and β (right) after exchanging the kinetics block (α_*K*_,β_*K*_, top) or nonlinearities (α_*N*_,β_*N*_, bottom) are plotted against the values of α_*c*_ and β_*c*_ in the control condition for eight On-Off amacrine and ganglion cells. The scale is logarithmic.

However, we cannot assume that the above effects of the nonlinearity and kinetics block will combine additively in the system. Thus we tested the source of differential adaptation in the two pathways by exchanging different components between the two pathways. We exchanged either the nonlinearities or kinetics blocks between pathways, and then measured the resulting effects on each pathway. First, we switched the kinetics blocks while keeping the filters and nonlinearities fixed, which caused a small change in the output of the two pathways as a function of contrast (Fig 7A). Then we exchanged the nonlinearities and saw a much larger effect, causing the On and Off pathways to swap their adaptive behavior.

We further quantified these effects by computing two parameters of the relationship between the contrast, *c* and output magnitude σ for each pathway. We computed the average slope α of σ(*c*), reflecting the average increase in output magnitude with contrast. If there was no adaptation, the standard deviation σ(*c*) would be proportional to the contrast with a slope of one, and so the more shallow slope of the Off pathway indicated more adaptation (Fig 7A). Then we fit an exponential function to σ(*c*), measuring the decay constant β for how fast σ(*c*) began to plateau with contrast. This indicates how rapidly adaptation occurred as contrast increased, with a smaller value indicating that adaptation developed at a lower contrast.

Fig 7B shows the values for the change in output α and rate of adaptation β for the two pathways in the control condition (α_*C*_,β_*C*_), when the kinetics blocks were exchanged (α_*K*_,β_*K*_) and when the nonlinearities were exchanged (α_*N*_,β_*N*_). In the control condition, the two pathways differed in the change in output α_*c*_ by a factor of 3.2 ± 0.2, with the On pathway having a greater slope reflecting less adaptation. Exchanging the kinetics caused the slope α_*K*_ to be only a factor of 1.3 ± 0.06 different than α_*C*_, a change of 30%. This factor was computed by averaging α_*C*(*ON*)_/α_*K*(*ON*)_ and α_*K*(*OFF*)_/α_*C*(*OFF*)_. In comparison, changing the nonlinearities caused the slope α_*N*_ to be a factor 2.94 ± 0.34 different from α_*c*_ in the control case, a change of 194%. Thus the change produced by exchanging the nonlinearities was 6.5 times that produced by exchanging the kinetics blocks. We then compared the control rate of adaptation β_*c*_ for the two pathways, which differed by a factor of 5.7 ± 0.9. Exchanging the kinetics blocks caused the rate β_*K*_ to be only a factor of 1.26 ± 0.07 different than β_*c*_, whereas exchanging the nonlinearity caused β_*N*_ to be a factor of 4.26 ± 0.72 different than β_*c*_. Thus, exchanging the nonlinearities caused a change in the adaptive rate 12.5 times greater than exchanging the kinetics. We conclude that the amount of adaptive change in temporal differentiation is controlled primarily by the different nonlinearities in the two pathways, with a smaller contribution from the different adaptive kinetics. In comparison, the contrast at which the adaptive change in the temporal filter occurs is controlled almost exclusively by the differences in the two nonlinearities.

This does not suggest that synaptic kinetics plays a small role in other adaptive properties. Across contrasts, the action of the kinetics block contributes to changing the speed of the temporal filter and the gain [18,20]. These effects, however are distinct from the change in temporal differentiation controlled by differential output in the two pathways.

## Discussion

Our results reveal a number of findings about contrast adaptation in On Off retinal ganglion cells, a system with multiple adaptive properties and mechanisms. First, adaptive changes in temporal bandwidth are generated in a manner distinct from changes in gain, the speed of the response and the mean response level. Second, changes in temporal bandwidth specifically require the On pathway, whereas other adaptive changes do not. Thus, we report a remarkably specific relationship between a mechanism, the On pathway, and one of multiple nonlinear properties of adaptation. Third, contrast causes the On and Off pathways to adapt to contrast differently, changing the ratio of On to Off input at high contrast. Because the On pathway has a more delayed average temporal filter than the Off pathway, a greater On pathway contribution results in a more biphasic filter for the entire system. Fourth, we verified using pharmacology and a model that On-Off ganglion cells under a uniform stimulus behave as though they have the computational structure of two independent adapting pathways whose outputs are summed together. This is a computational conclusion, and the true circuit structure may differ. Finally, analysis of that model indicates that the key component that causes differential adaptation between the On and Off pathways is a difference in threshold, rather than differences in nonlinear dynamics. This implies that the differences in the threshold of the bipolar cell terminal as compared to the baseline membrane potential are responsible for the change in temporal bandwidth, as opposed to differences in the dynamics of synaptic release in the two pathways.

We have used pharmacology to block the signals traveling through one receptor type, and then infer the independent contributions of the blocked and unblocked pathways. It is important to note that the subtractive procedure we have performed to make this inference is not generally valid unless the two pathways do sum together independently. Thus, the use of the LNK model to verify this computational structure, and pharmacology to validate this aspect of the model is a necessary step in the interpretation of the two summed pathways. Crossover inhibition between the On and Off pathways is observed in many ganglion cells [29]. However, when we fit a two-pathway LNK model in the control condition, the Off pathway of the model captures the cell’s behavior when only the Off pathway is active under APB. The model’s symmetric structure lends support to the idea that blocking the On pathway can reveal the contributions of both Off and On pathways. Note that the conclusion that the cells conform to a model of two independently adapting pathways does not mean there is no crossover inhibition. The two pathways consist of one pathway that originates from APB sensitive neurotransmission, and one that does not. The APB sensitive pathway may itself be an average of several neural pathways, some that deliver a signal to some component of the Off pathway.

One reason, however, to think the effect of crossover inhibition is not strong in On-Off ganglion cells is that crossover inhibition often creates a more linear cell [38], whereas On Off cells are necessarily nonlinear, and have a sharp threshold. A second effect is for the On pathway to create a more strongly rectifying Off pathway by shifting the effective threshold of the Off pathway through tonic inhibition [39]. Because the same Off pathway in the model fit the response with and without the On pathway present, we predict similarly that this effect of crossover inhibition is not strong here. If there is crossover inhibition in these cells, it may contribute to the initial linear filter in each pathway, which mechanistically would correspond to presynaptic inhibition onto the bipolar cell terminal. The majority of salamander On-Off ganglion cells have direct input from both On and Off bipolar cells, with a smaller percentage (< 25%) having input from one pathway that is delivered only via intervening amacrine cells [40]. This more common type might be expected to behave according to the model of independently adapting pathways we describe here. We cannot assume, however, that On and Off pathways are strictly parallel streams that do not interact, and we expect this will change with more complex visual stimuli than the uniform field stimuli used to create the model. Nonetheless, the basic conclusion that the On pathway is required for large changes in temporal frequency but not other adaptive properties is observed with a spatiotemporal stimulus (Fig 2).

An alternative computational structure has been proposed to account for contrast adaptation in ganglion cells using a divisive effect of one visual feature on another [24]. This Div-S model can capture fast adaptation in On ganglion cells of the mouse, but does not include slow adaptive time constants and thus was not tested here. In the LNK model, the threshold of the nonlinearity plays a critical role, such that a higher contrast increases the mean of the input to the kinetics block, which then adapts. Thus a strongly rectified synaptic terminal would experience synaptic depression when the contrast increases. Because of the necessity of the threshold, this arrangement may be less effective at generating adaptation for a more linear cell. Because the Div-S model has been shown to fit On ganglion cells, which are more linear than Off cells [36,37], it may be that a divisive structure is required for adaptation in more linear cells.

### Differential adaptation in parallel pathways

Systems that can be accurately captured with a linear-nonlinear (LN) model do not change their properties of temporal filtering or sensitivity with a change in stimulus statistics. Thus, a change in the parameters of the LN model has served as a definition of adaptation [7,26,41,42]. Both On and Off pathways adapt independently, in that the gain of each pathway changes with contrast [20]. Overall, the Off pathway adapted at a lower contrast, and reached a higher level of adaptation at lower contrasts than the On pathway (Fig 6A). This change in the relative strength of the On and Off pathways is consistent with an earlier study [43] that showed that one type of On-Off ganglion cell–type II from that study–changed its temporal filter with contrast, but this behavior could not be reproduced by a single LNK pathway. This earlier study identified different clusters in a spike-triggered covariance analysis that could have been generated by the On and Off pathways, although the small amount of spikes occurring from the On pathway was insufficient to model. Our membrane potential recordings here provided sufficient data to fit a two-pathway LNK in order to analyze the internal computational properties of the separate On and Off input. Although the different adaptive kinetics do make a small contribution to differential adaptation, the primary source for this adaptive change is the different thresholds in the two pathways (Figs 5 and 6). Thus in general, changes in temporal bandwidth alone could potentially be produced through the summation of non-adapting (LN) pathways.

### Differences in On and Off inputs

A number of studies in different species have shown that On and Off pathways have differences in their nonlinear properties. On ganglion cells are more linear and have lower thresholds in salamander, mouse and primate [36,37,44], and On pathway neurons are similarly more linear in the fly early visual system [45]. Yet we observe that for On-Off ganglion cells, On pathway input has a higher threshold (Fig 3). There is substantial diversity in the responses of individual bipolar cells in salamander [40,46] and mouse [47], and it may be that On bipolar cell inputs to On-Off ganglion cells have a higher synaptic threshold than On bipolar cell inputs to On type ganglion cells.

### Role of adaptive synapses

Properties of synaptic vesicle release have been shown to produce all types of adaptive properties discussed here. Depletion of synaptic vesicles can cause changes in gain, the elevated rate constants of release as a function of high calcium concentration can speed the overall system dynamics, and slow replenishment can create a homeostatic effect on the average response. Synaptic depression could also potentially cause some level of a change in temporal bandwidth, in that at high release rate, synaptic depression can create a more transient response, thus attenuating low temporal frequencies. Thus, although all types of observed adaptive changes could potentially be carried out by synapses in a single pathway, the large shift in the temporal bandwidth we measure here required a summation of two neural pathways that adapt differently to contrast. Because the effect of the dynamics of synaptic vesicle release, and the flexibility of the effect of the rate constants on adaptive behavior, one might think that the different adaptive dynamics in the On and Off pathways could arise from differences in synaptic release. Our computational analysis, however, indicates that the higher threshold of the On pathway in On-Off cells, which has previously been reported, gives rise to that differential adaptation.

The LNK model breaks down the cell’s response into smaller subsystems, first by separating On and Off pathways, then further compartmentalizing computational function into feature selection, nonlinear distortion and thresholding, and adaptation. The accuracy of this model, its ability to capture adaptive properties and the correspondence of its components to distinct neural pathways (Fig 3) allow us to localize a potentially complex response into simpler computational elements. In the model, as the variance of the signal increases, the mean of the signal after the threshold will increase. This increase in mean then triggers adaptation of the kinetic system. Thus, one can think of the threshold as being a contrast threshold for adaptation It is this threshold that is different between the two pathways, although the kinetic system that implements that adaptation is similar between the pathways. This similarity of adaptive properties is consistent with the notion that blocking the On pathway does not change gain, speed or offset. If the two adaptive pathways were different one would expect that changing the mixture would change adaptation of the overall system.

Different types of mechanisms can generate changes in temporal differentiation. Photoreceptors have a more biphasic response at higher luminance [48], yet use only a single biochemical pathway to convey the light response [49]. Insect photoreceptors accomplish this switch with a combination of two ionic conductances with different thresholds [50]. Previous experiments give evidence as to the correspondence of different stages of the LNK model with different levels in the circuit. Because the bipolar cell membrane potential does not show strong rectification for a constant mean intensity stimulus but ganglion cells are rectified, the threshold likely arises at the bipolar cell synaptic terminal from voltage-activated calcium channels. One possible source of the different thresholds in the two pathways is differential expression of such calcium channels, which differ in rods in cones. It is unknown, however, whether they differ in On and Off bipolar cells [51]. An alternative source is differential inhibition onto bipolar cell terminals [52], which would set the resting potential at different levels relative to the activation threshold of calcium channels in the synaptic terminal.

### Two pathways implement a change in the rules of efficient coding

The rules of efficient coding are known to change in a sophisticated way when the signal strength changes. Due to the dominance of low temporal frequencies in natural visual scenes, it can be an efficient strategy to remove these slow correlations, thus giving more equal weight to high and low frequency signals [5,9]. This approach however can come at a cost when signals are weak and noisy–rather than computing the difference between noisy signals, the more efficient strategy is not to decorrelate, but to simply exclude high frequency signals. Here we see that this shift in temporal frequency in On- Off ganglion cells comes mostly from a change in the activation of one neural pathway to two pathways.

The switch from one to two neural pathways is highly similar to that found in the auditory cortex, where the stimulus-response relationship changes from monophasic to biphasic at higher signal strength [53]. In that case, there is an accompanying switch from a one-dimensional to a two-dimensional stimulus representation. However, because of a lack of knowledge of neural inputs in the cortex it is not known whether this change involves the additional contribution from a separate neural pathway as we can conclude in the retina.

Our results show that distinct properties of contrast adaptation are controlled by distinct mechanisms acting in concert, with the baseline set of properties of changing gain speed and offset present in individual neural pathways, and temporal bandwidth changing arising from the recruitment of a high threshold pathway. Thus, the system is layered, with a baseline rule of efficient coding implemented in single pathways, and a second-level rule selected or rejected by the high threshold of a second pathway. Different rules of efficient coding are selected by different combinations of neural pathways.

## Methods

Electrophysiology. This study was performed in strict accordance with the recommendations in the Guide for the Care and Use of Laboratory Animals of the National Institutes of Health, and the Stanford institutional animal care and use committee (IACUC) protocol (11619). For intracellular recording, the intact salamander retina of either sex was held in place under a transparent dialysis membrane containing several 150–300 μm holes. Intracellular electrodes were filled with 2 M potassium acetate (200–300 MΩ) and guided into the retina under infrared illumination viewed through a CCD camera. Bipolar cells, On-Off amacrine or ganglion cells were identified by their flash response and level in the retina. Ganglion cells were recorded in the ganglion cell layer, and On-Off amacrine cells were recorded in the inner nuclear layer. Intracellular recordings had resting membrane potentials more hyperpolarized than– 65 mV, and were made from eight On-Off cells including four ganglion cells and four amacrine cells. Results were similar for the two cell types, and are pooled in all analyses. Recordings overall ranged from 10–90 minutes in duration.

Visual Stimulation. A spatially uniform visual stimulus was projected from a video monitor onto the retina. The stimulus intensity changed every 30 ms, and was drawn from a Gaussian probability distribution with constant mean intensity, *M* (~ 8 mW/m^2^) and standard deviation *W* [54]. Contrast was defined as *W*/*M*, and changed randomly every 20 s to a value between 0.05 and 0.35, drawn from a uniform distribution. The stimulus lasted 300 s (15 contrast levels) and the identical stimulus sequence was repeated at least two times.

Standard Linear-Nonlinear (LN) model. A linear temporal filter was computed that most closely approximates the entire response of a cell or pathway. This filter *F*_*LN*_(*t*) is different than the component linear filter of the LNK model, *F*_*LNK*_(*t*). Linear temporal filters were computed as described [7]. The stimulus intensity *s*(*t*) was normalized to have zero mean, and a standard deviation equal to the contrast. The filter, *F*_*LN*_(*t*), was computed as the correlation between *s*(*t*) and the response *r*(*t*) normalized by the autocorrelation of the stimulus. The Fourier transform of the filter was computed as,

$${\tilde{F}}_{LN}\left(\omega \right)=\frac{\langle {\tilde{s}}^{*}\left(\omega \right)\tilde{r}\left(\omega \right)\rangle }{\langle {\tilde{s}}^{*}\left(\omega \right)\tilde{s}\left(\omega \right)\rangle }$$ (1)

where $\tilde{s}\left(\omega \right)$ is the Fourier transform of *s*(*t*), ${\tilde{s}}^{*}\left(\omega \right)$ its complex conjugate, and 〈…〉 denotes averaging over 1 s segments spaced every 0.1 s throughout the recording. The denominator corrects for deviations of the video monitor from a white noise distribution [55]. This calculation was performed separately at low (5%) or high (35%) contrast. The filter was normalized in amplitude so that when the filter was convolved with the stimulus to yield a linear prediction *g*(*t*),

$$g(t)=\int F_{LN}(t-\tau )s(\tau )d\tau ,$$ (2)

the variance of *g*(*t*) and *s*(*t*) were equal,

$$\int s^{2}\left(\tau \right)d\tau =\int g^{2}\left(\tau \right)d\tau$$ (3)

Linear Nonlinear Kinetic model. LNK models were optimized as described, [20], where additional details can be found. For On-Off cells the model had two pathways. Each pathway consisted of a linear temporal filter, a static nonlinearity, and a first order kinetic system. The components were parameterized as described below, and all parameters were fit together using a constrained optimization algorithm. For each pathway, the stimulus, *s*(*t*), was passed through a linear temporal filter, *F*_*LNK*_(*t*) and a static nonlinearity, *N*_*LNK*_(*g*),

$$u(t)=N_{LNK}\left(\int F_{LNK}(t-\tau )s(\tau )d\tau \right).$$ (4)

Although these two initial stages have the same structure linear-nonlinear (LN) model, the filter and nonlinearity are different functions than those computed for an LN model, and are optimized, rather than computed using reverse correlation. As in [20], the nonlinearity was a sigmoid function parameterized as:

$$N_{LNK}(x)=a^{\left(\mathrm{erf}\left(\kappa x+b_{1}\right)+1\right)}k_{a}^{-1}+b_{2}$$ (5)

where erf(*x*) is the error function defined for cumulative Gaussian distributions, *a*, *b*_1_ and *b*_2_ are parameters of the model, and *κ* was a constant equivalent to the overall variance of the filtered stimulus *g*(*t*). The exponent of *a* varies between zero and twenty, and the activation rate constant *k*_*a*_ in the denominator scales the nonlinearity so that its maximum is one. Thus, the output of the nonlinearity, *u*(*t*), scales the activation rate in the kinetics block between zero and *k*_*a*_.

The kinetics block of the model is a Markov process defined by

$$\frac{dP^{T}\left(t\right)}{dt}=P^{T}\left(t\right)Q\left(u\right),$$ (6)

where **P**(*t*) is a column vector of *m* fractional state occupancies such that $\sum_{i}P_{i}=1$, and **Q** is an *m*×*m* transition matrix containing the rate constants Q_*ij*_ that control the transitions between states *i* and *j*, with $Q_{ii}=-\sum_{i\neq j}Q_{ij}$. After this differential equation was solved numerically, the output of the model, *r*′(*t*) was equal to one of the state occupancies scaled to a response in millivolts,

$$r^{\prime }\left(t\right)=P_{2}\left(t\right)c+d,$$ (7)

where *c* and *d* are a scaling and offset term for the entire recording.

States and rate constants are diagrammed in Fig 2A and defined as,

$$\begin{array}{llll} P_{1}=R & \mathrm{Resting} & Q_{12}=u\left(t\right)k_{a} & \mathrm{Activation}\\ P_{2}=A & \mathrm{Active} & Q_{23}=k_{fi} & \mathrm{Fast}\;\mathrm{inactivation}\\ P_{3}=I_{1} & \mathrm{Inactivated} & Q_{31}=k_{fr} & \mathrm{Fast}\;\mathrm{recovery}\\ P_{4}=I_{2} & \mathrm{Inactivated} & Q_{34}=k_{si} & \mathrm{Slow}\;\mathrm{inactivation}\\ & & Q_{43}=u\left(t\right)k_{sr} & \mathrm{Slow}\;\mathrm{recovery} \end{array}.$$ (8)

The change in state occupancy was thus determined as

$$\frac{dP^{T}\left(t\right)}{dt}=\left(\begin{array}{c} {\dot{P}}_{1}\left(t\right)\\ {\dot{P}}_{2}\left(t\right)\\ {\dot{P}}_{3}\left(t\right)\\ {\dot{P}}_{4}\left(t\right) \end{array}\right)=P^{T}\left(t\right)\left(\begin{array}{cccc} -u\left(t\right)k_{a} & u\left(t\right)k_{a} & 0 & 0\\ 0 & -k_{fi} & k_{fi} & 0\\ k_{fr} & 0 & -\left(k_{fr}+k_{si}\right) & k_{si}\\ 0 & 0 & u\left(t\right)k_{sr} & -u\left(t\right)k_{sr} \end{array}\right).$$ (9)

The output of the two LNK pathways were then scaled with independent weights *w*_*ON*_ and *w*_*OFF*_ and summed to give the final output of the model. As previously described [20], a k-fold cross validation method was performed using data divided into five sets consisting of interleaved 300 ms segments. The model was optimized on four data segments and tested on the fifth. Quoted cross-correlation values are the average across all five segments. Unless otherwise stated, error bars indicated s.e.m.

## Supporting information

S1 Fig Intracellular recordings from ganglion cells at different contrasts. **A.** Two superimposed traces of the flash response from an example On-Off ganglion cell. Bar indicates the time of light On. **B.** Intracellular recording of a ganglion cell responding to two repeated presentations of the same white noise stimulus sequence consisting of a uniform field at high contrast (35%). Also shown is the subthreshold membrane potential of one of these repeats extracted for further analysis. **C.** Same as **B** for the transition (dotted line) from high (28%) to low (5%) contrast. A slow recovery from hyperpolarization can be seen in the 5% contrast traces.
(PDF)

S2 Fig Linear-nonlinear models showing adaptation in the ganglion cell spiking response. **A.** Spatiotemporal filter of an example On-Off ganglion cell. **B.** LN models from the cell responding to high (35%) and low (5%) contrast in a control condition (top) and in the presence of APB (bottom). The average time course of the cell is shown computed as the first principal component of the spatiotemporal filter.
(PDF)

S3 Fig Adaptive changes in gain, delay and offset do not correlate with On pathway input. **A.** Gain change between high and low contrast for ganglion cell spiking shown as a function of the On/Off ratio of each cell. Results shown in a control condition (solid symbols and lines) and with APB (open symbols and dotted lines). Lines are fits to the data. **B.** Change in the time to the first negative peak between high and low contrast shown as a function of the On/Off ratio of each cell. **C.** Slow offset during adaptation to high or low contrast as a function of the On/Off ratio of each cell.
(PDF)

S4 Fig Bipolar cells do not change their temporal frequency during contrast adaptation. **A.** Two superimposed traces of the flash response from an example Off bipolar cell. Bar indicates the time of light On. **B.** LN model for bipolar cells responding to a uniform field stimulus as in Fig 1 that changed every 20 s to a new contrast ranging between 5–35%. **C.** Median temporal frequency computed from the linear temporal filter, as a function of contrast, averaged over all cells. **D.** Normalized gain as a function of contrast, averaged over six bipolar cells.
(PDF)
