# Supplementary figures and images for: Adaptive feature detection from differential processing in parallel retinal pathways
Source: PLoS Comput Biol. 2018 Nov 20;14(11):e1006560. doi: 10.1371/journal.pcbi.1006560 (PMC6245510; doi:10.1371/journal.pcbi.1006560)

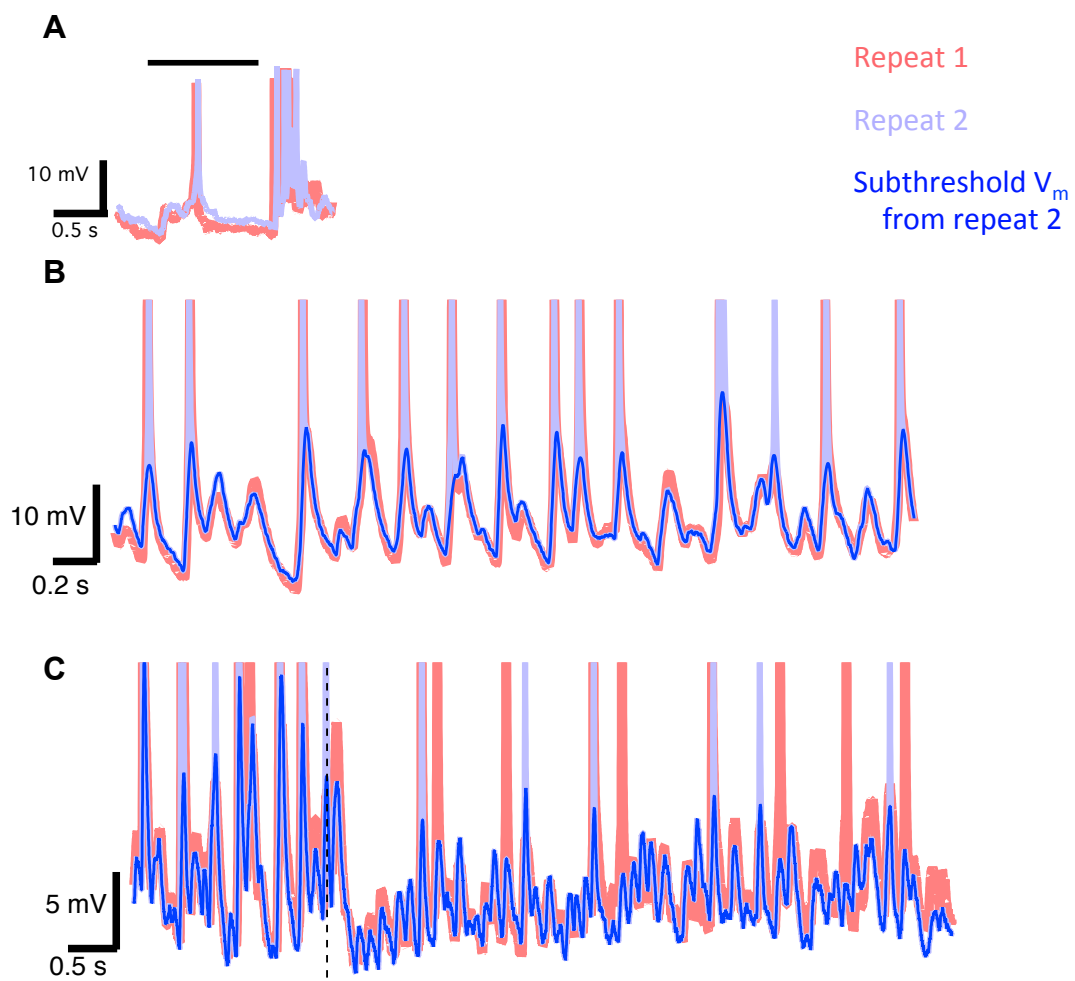

Supplement: S1 Fig — A. Two superimposed traces of the flash response from an example On-Off ganglion cell. Bar indicates the time of light On. B. Intracellular recording of a ganglion cell responding to two repeated presentations of the same white noise stimulus sequence consisting of a uniform field at high contrast (35%). Also shown is the subthreshold membrane potential of one of these repeats extracted for further analysis. C. Same as B for the transition (dotted line) from high (28%) to low (5%) contrast. A slow recovery from hyperpolarization can be seen in the 5% contrast traces. (PDF) [file pcbi.1006560.s001.pdf]

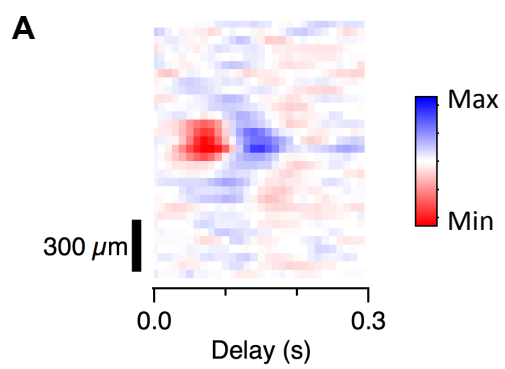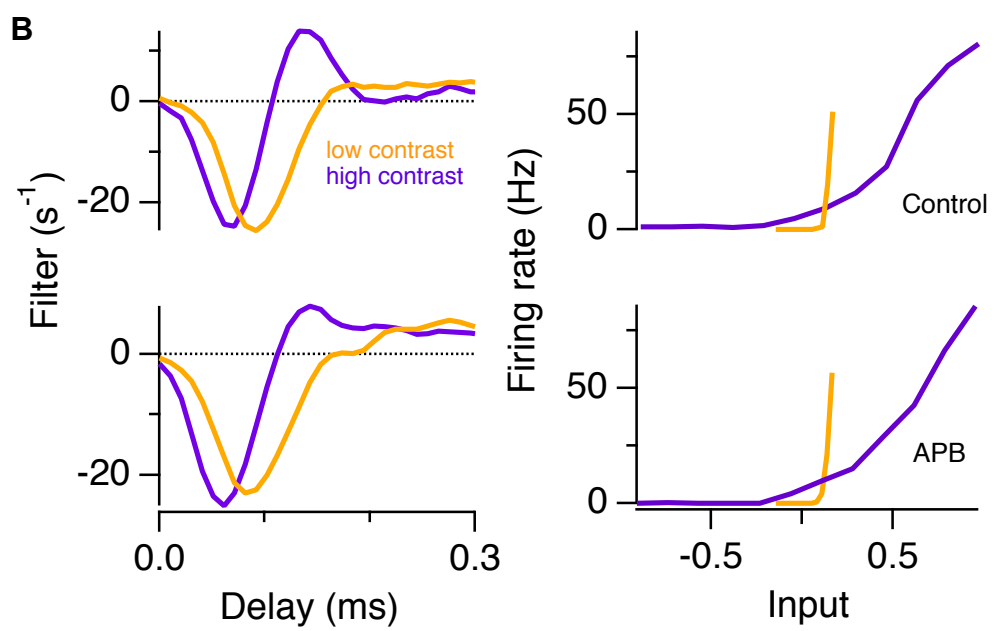

Supplement: S2 Fig — A. Spatiotemporal filter of an example On-Off ganglion cell. B. LN models from the cell responding to high (35%) and low (5%) contrast in a control condition (top) and in the presence of APB (bottom). The average time course of the cell is shown computed as the first principal component of the spatiotemporal filter. (PDF) [file pcbi.1006560.s002.pdf]

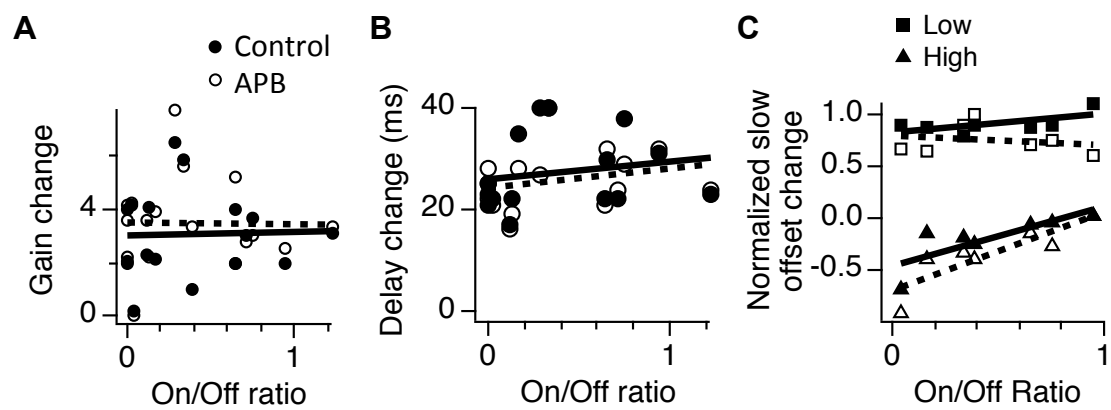

Supplement: S3 Fig — A. Gain change between high and low contrast for ganglion cell spiking shown as a function of the On/Off ratio of each cell. Results shown in a control condition (solid symbols and lines) and with APB (open symbols and dotted lines). Lines are fits to the data. B. Change in the time to the first negative peak between high and low contrast shown as a function of the On/Off ratio of each cell. C. Slow offset during adaptation to high or low contrast as a function of the On/Off ratio of each cell. (PDF) [file pcbi.1006560.s003.pdf]

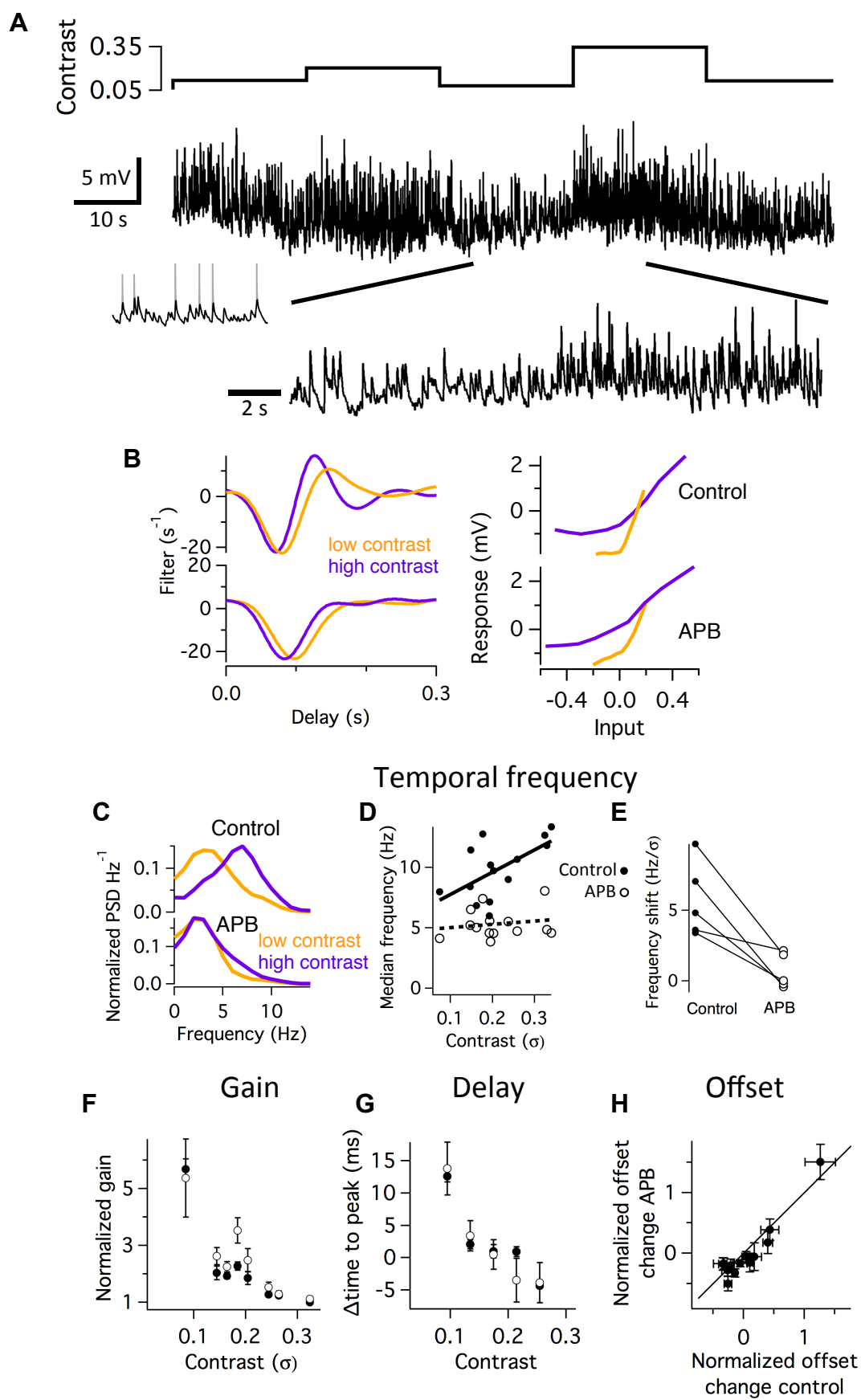

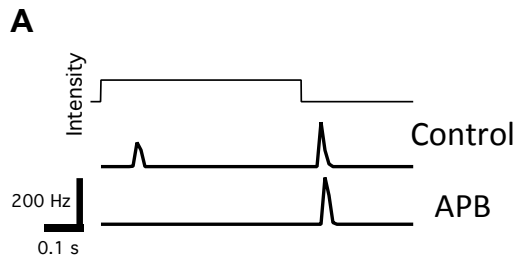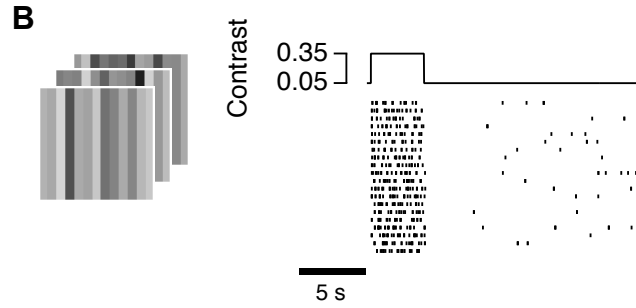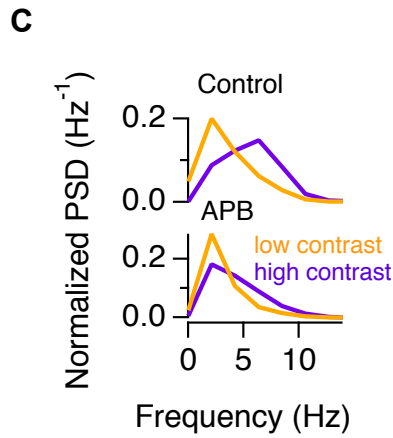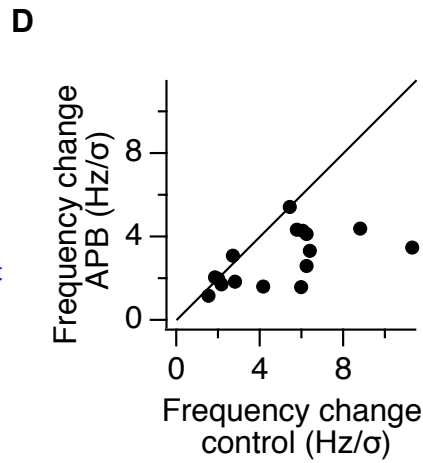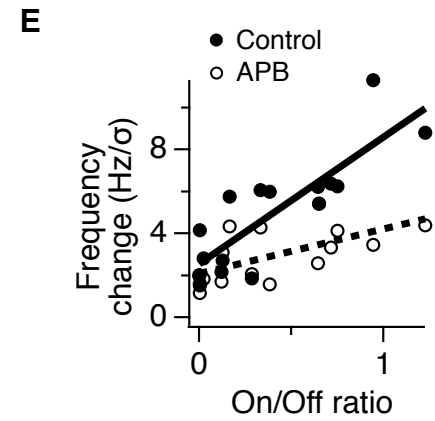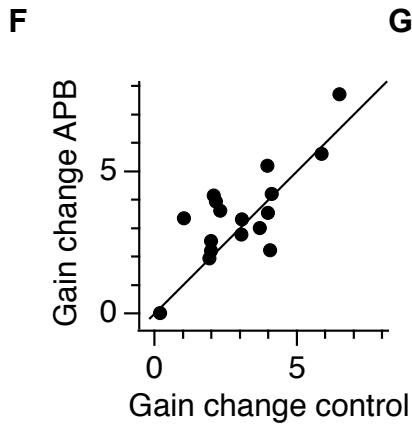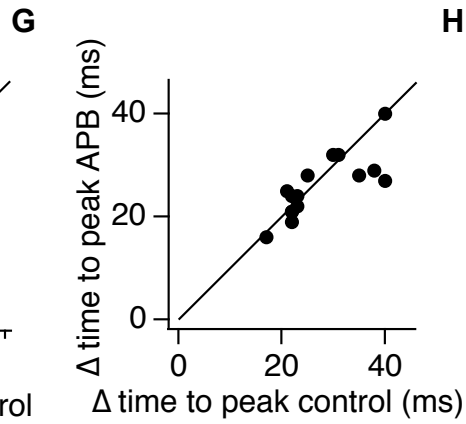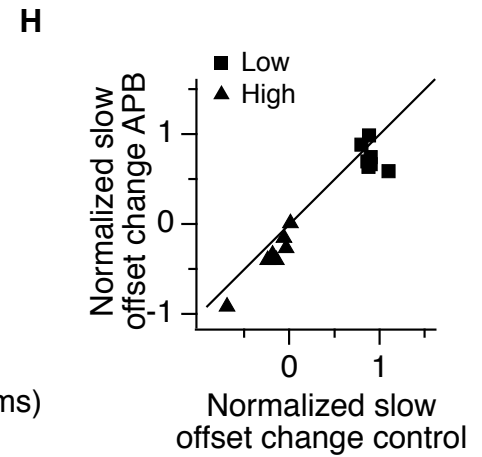

**A**

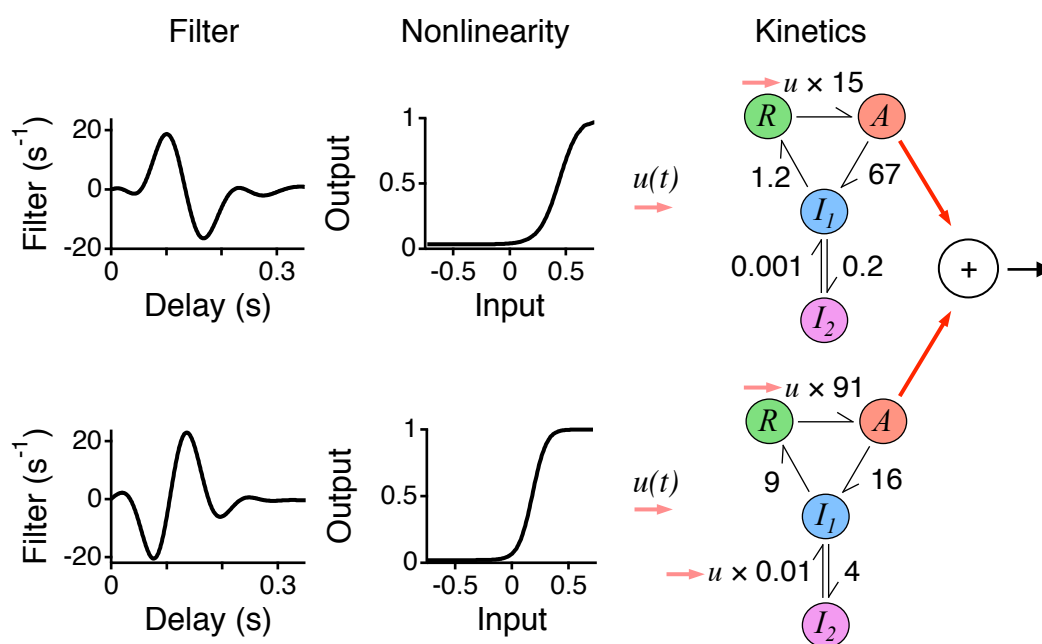

**B**

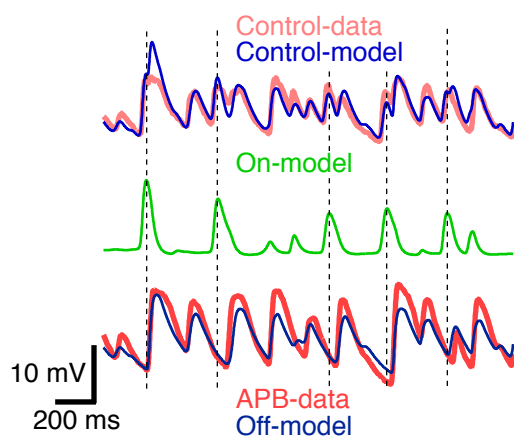

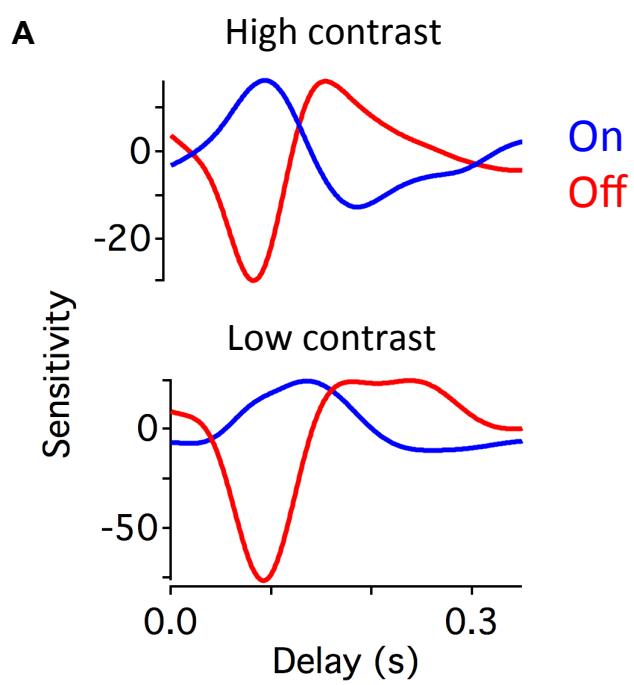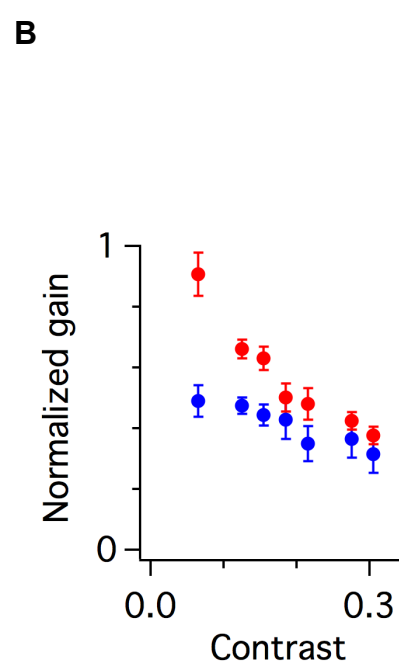

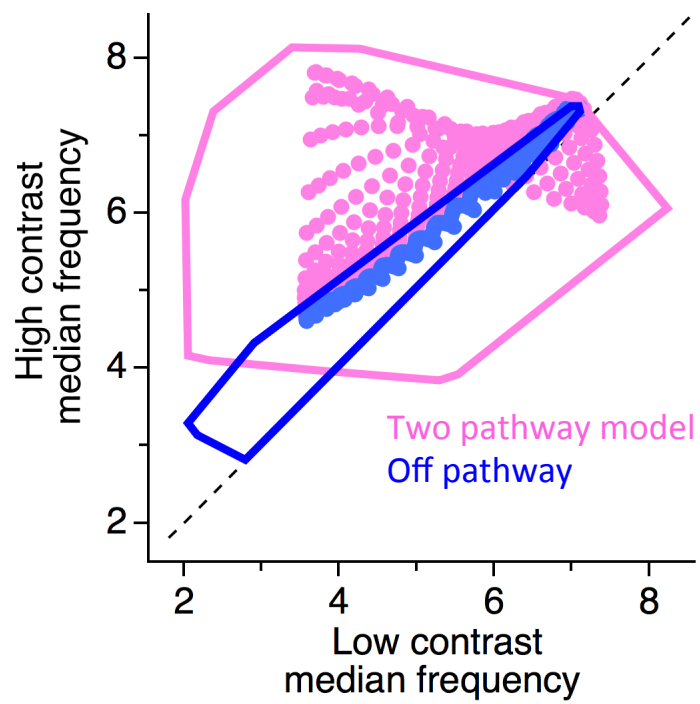

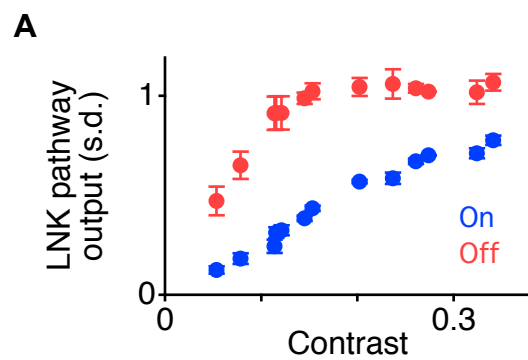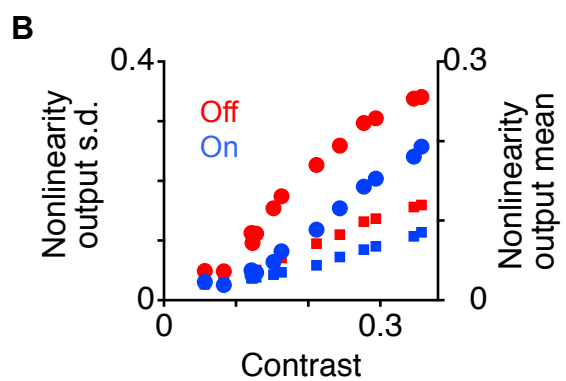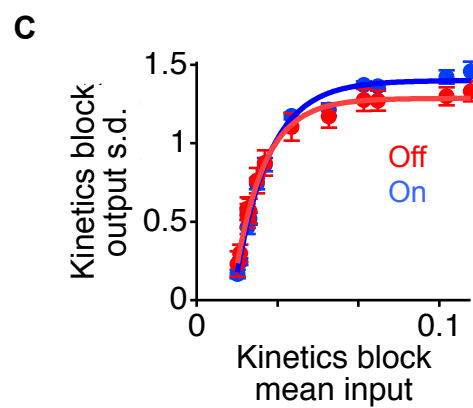

**A**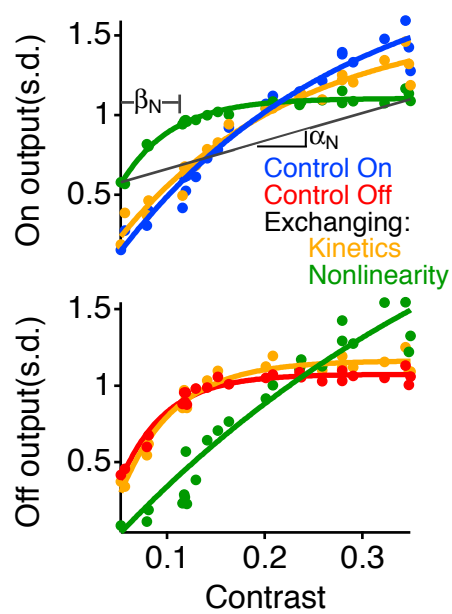**B**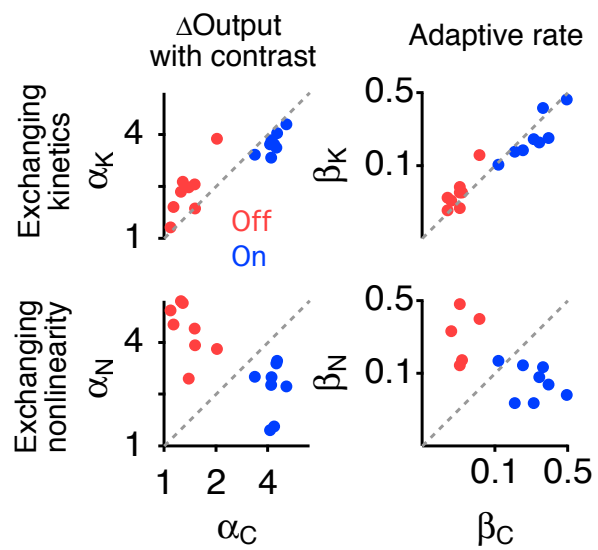

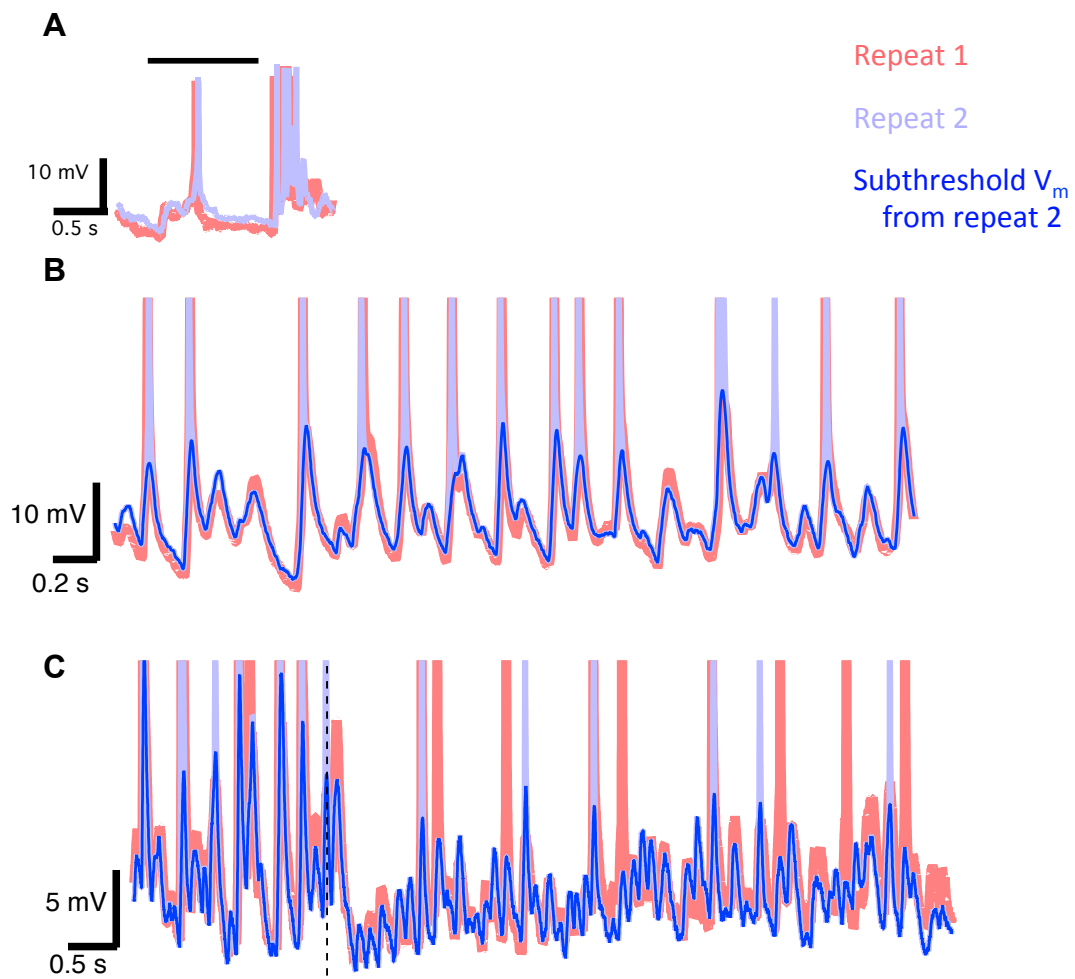

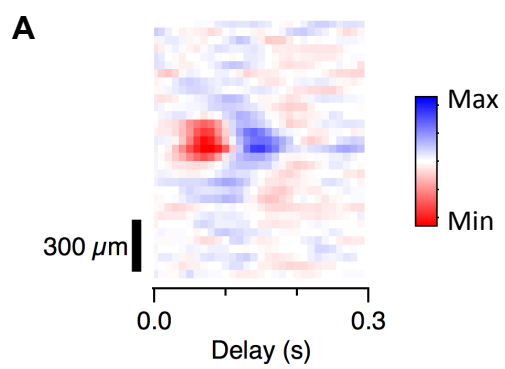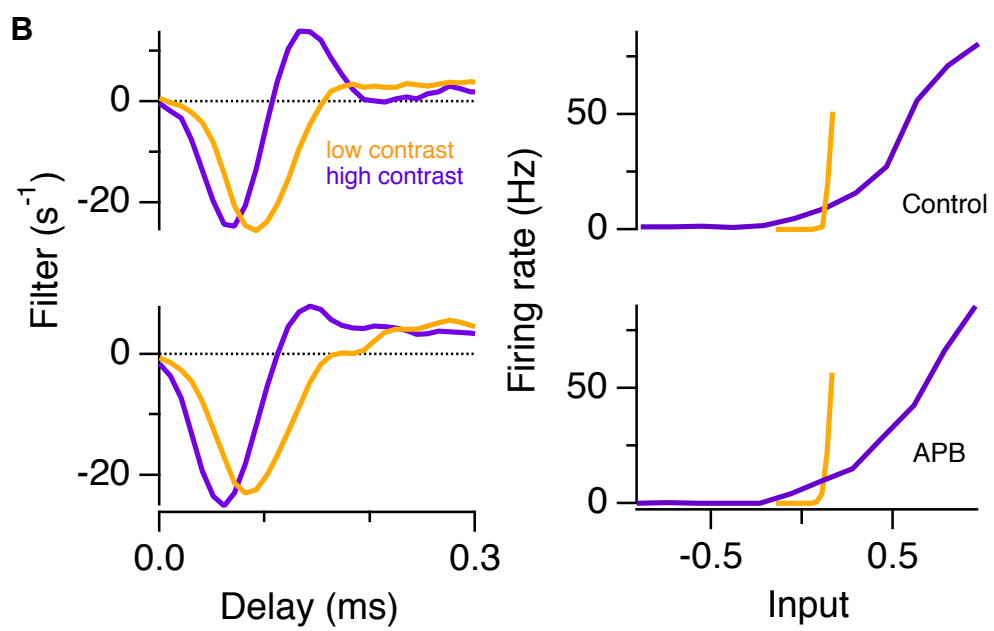

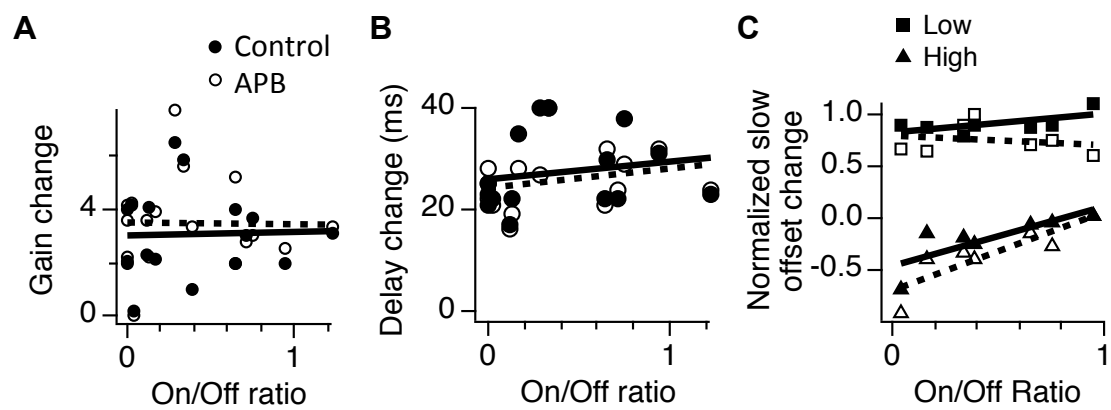

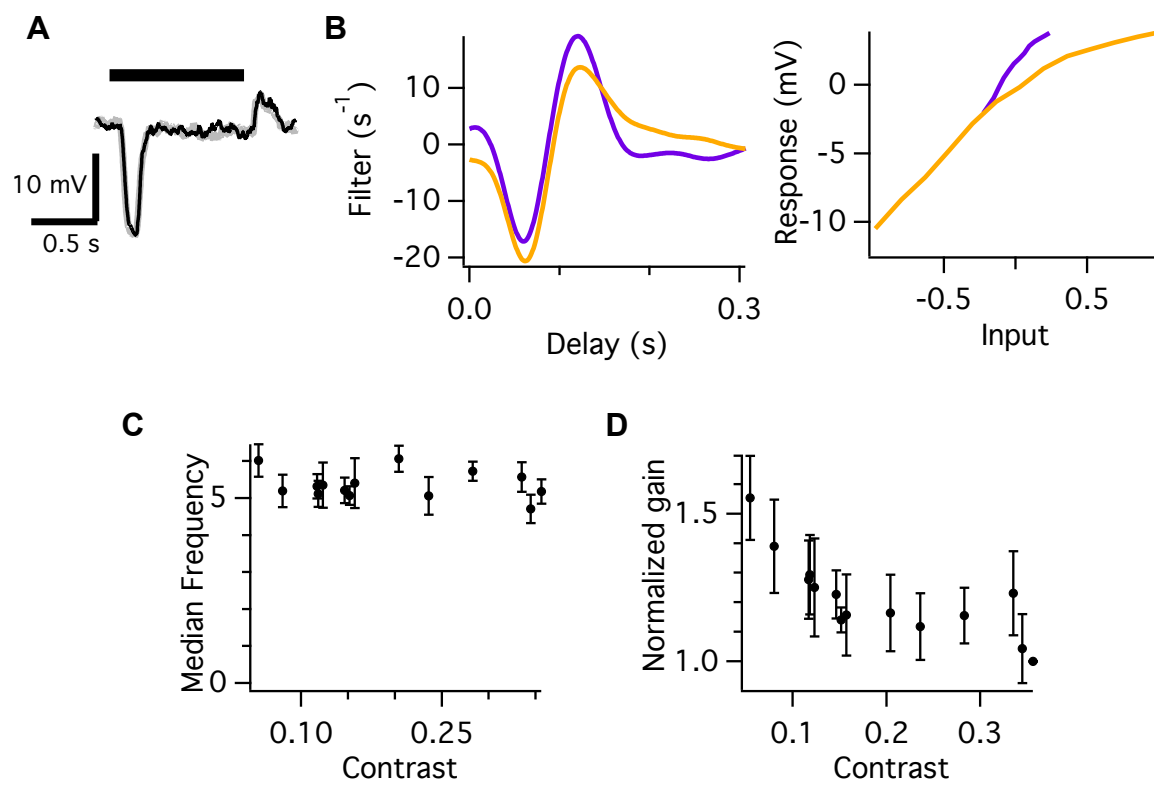

Supplement: S4 Fig — A. Two superimposed traces of the flash response from an example Off bipolar cell. Bar indicates the time of light On. B. LN model for bipolar cells responding to a uniform field stimulus as in Fig 1 that changed every 20 s to a new contrast ranging between 5–35%. C. Median temporal frequency computed from the linear temporal filter, as a function of contrast, averaged over all cells. D. Normalized gain as a function of contrast, averaged over six bipolar cells. (PDF) [file pcbi.1006560.s004.pdf]
